# Stimuli-Responsive Smart Polymer: A Precise Era with Artificial Intelligence and Machine Learning

**DOI:** 10.1021/prechem.5c00375

**Published:** 2026-03-27

**Authors:** Tamanna Pradhan, Subhajit Das, Khokan Mondal, Subrata Dolui

**Affiliations:** † Department of Chemistry, Indian Institute of Technology Kharagpur, Kharagpur, West Bengal 721302, India; ‡ Electronics and Communication Engineering (ECE), Institute of Engineering and Management, University of Engineering and Management, Kolkata, West Bengal 700160, India; § Department of Computer Science and Business System (CSBS), 471648 Techno India University, Second Floor EM-4, Sector-V, Salt Lake, West Bengal, Kolkata 700091, India; ∥ Department of Chemistry and Biochemistry, Concordia University, Montreal, Quebec, Canada H4B 1R6; ⊥ Department of Pharmaceutical Chemistry, School of Pharmacy, Rungta International Skills University, Bhilai 490024, Chhattisgarh, India

**Keywords:** Smart polymers, artificial intelligence, deep learning, data-driven materials design, inverse materials engineering, predictive informatics

## Abstract

Artificial intelligence (AI) and machine learning (ML)-based solutions are gaining popularity in the field of accelerated material design and development. In this review, we assess AI/ML-enabled advances in stimuli-responsive polymer informatics, with a focus on developing application-specific, practical polymeric materials. We look at exemplary design techniques in several critical and developing application areas, including materials design for energy production, storage, and conservation, as well as enabling a sustainable economy based on recyclable and/or biodegradable polymers. Early demonstrations demonstrated that the ML can accurately predict critical polymer characteristics and phase behavior, accelerating the discovery of high-performance compositions and optimizing processing conditions for stimuli-responsive hydrogels, thermosets, and 3D/4D printed smart structures. Integrating AI-assisted modeling with real-time sensing in smart polymers allows for closed-loop control over actuation, shape morphing, drug release, and solubility/swelling changes in complicated, time-varying environments. The combination of stimuli-responsive polymeric smart materials with AI/ML-driven design and control enables programmable, application-specific smart materials that correspond with precision chemistry’s goal.

## Graphical abstract

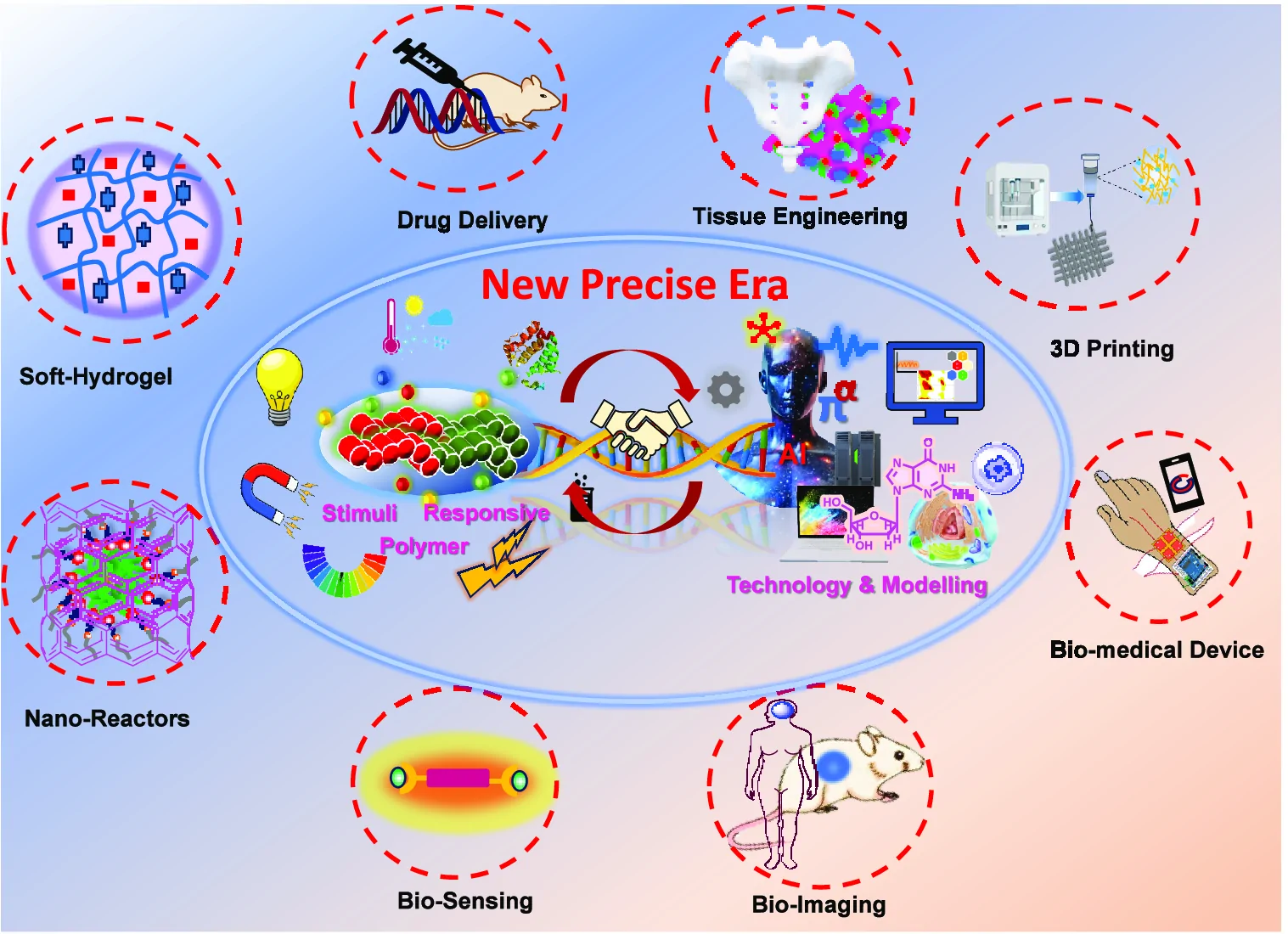

## 1 Introduction

Material innovation has been a fundamental driving force in human civilisation. Throughout human cultural history, functions have been discovered and designed for everything from matter to materials, and they continue to have an impact on modern life and the future. The pursuit of knowledge from data is linked to materialism and may be traced back to *Homo sapiens*’ “keep records by tying knots” and the Pythagoreans. “All things are numbers” mentality. “Smart” stimuli-responsive polymers can respond to variations in the external environment, including light, redox, temperature, pH, and ionic strength. Because of their broader potential applications, these polymers have gained interest to both biomedical science and engineering.^1,2^ The presence of functionality in the polymer chain controls how these polymers react to stimuli. The production of dual- or multistimuli responsive polymers may lead to the development of novel, complex applications in contrast to polymers that can react to single stimuli. This has prompted a great deal of research and the synthesis of more advanced synthetic instruments to create these multiresponsive polymers.^3,4^ Recent advances of different reversible deactivation radical polymerization (RDRP) techniques have facilitated the synthesis of novel amino acid stimuli-responsive polymers. This progress is largely attributed to the development of novel polymerization techniques for functional monomers. Different RDRP techniques such as single electron transfer-living radical polymerization (SET-LRP),^5–7^ atom transfer radical polymerization (ATRP),^8,9^ photo RDRP,^10^ electrochemically mediated atom transfer radical polymerization (eATRP),^11^ organometallic-mediated radical polymerization (OMRP),^12^ reversible addition–fragmentation chain transfer (RAFT) polymerization,^13^ organoheteroatom-mediated radical polymerization,^14^ nitroxide-mediated radical polymerization (NMP)^15^ and iodine transfer polymerization (ITP)^16^ are being utilized to synthesize unique well-defined (co)­polymers.

Over the years, research has been inspired by nature and has experimented with developing synthetic materials generated from biomolecules that resemble natural proteins in both structure and function. Those materials are excellent prospects for nonfouling surfaces, targeted drug and gene delivery, self-assembling nanocarriers, and regenerating tissue scaffolds because they have innate biocompatibility, programmable self-assembly, tunable degradation, and precise locations for functional modification. Recent reviews^17–20^ highlight the variety of smart polymer architectures—hydrogels, self-healing networks, shape memory systems, and dye-functionalized polymers—engineered to respond to stimuli such as pH, temperature, light, redox environment, and biomolecular signals. However, navigating the vast compositional and processing design space to accurately adjust responsiveness, multistimuli coupling, and device-level performance remains a major barrier. AI/ML frameworks, including supervised learning, deep neural networks, Bayesian optimization, and generative models, are being used to identify structure–property connections, anticipate transition thresholds, and direct autonomous or semiautonomous synthesis cycles in polymer systems.^21,22^ Scalable, repeatable production has been made possible by recent developments in peptide and polymer synthesis, which have increased the available sequence space. Discovery procedures are being revolutionized at the same time by AI/ML. Data-driven algorithms expedite the mapping of sequence-property connections, forecast drug-release profiles, direct inverse design, and rank candidates. ML finds nonintuitive design guidelines and reduces development time by eliminating the need for laborious trial-and-error. Small and diverse data sets, uneven reporting of composition, processing, degradation, and biocompatibility criteria, and a lack of interoperable high-throughput assays are the field’s main constraints. Interpretable models that link mechanism to prediction and validation pipelines that guarantee safety and manufacturing viability are crucial. In this review, we summarize the AI/ML field in the context of stimuli-responsive polymers and their current state of the art. We cover the development of stimuli-responsive polymer materials from 1995 onward, and the application of these materials in the AI/ML field from 2018 to 2025. Bejagam et al.^23^ reported that the thermoresponsive poly­(*N*-isopropylacrylamide (PNIPAM) coil–globule transition may be quantitatively resolved using machine-learning-assisted coarse-grained modeling, offering molecular-level insight into LCST behavior in addition to empirical fitting. Kumar et al.^24^ demonstrated inverse machine-learning design of cloud-point temperatures in poly­(2-oxazoline) systems utilizing regression models combined with optimization techniques, allowing for tailored thermoresponsive polymer manufacturing. Boztepe et al.^25^ used artificial neural networks (ANNs) to model dual pH- and temperature-responsive poly­(*N*-isopropylacrylamide-*co*-acrylic acid)/poly­(ethylene glycol) (poly­(NIPAAm-*co*-AAc)/PEG) IPN hydrogels. They accurately predicted doxorubicin release profiles under combination stimuli. Cobb et al.^26^ compared ANN, random forest, SVR, and elastic-net models to predict the transition temperatures (Tt) of elastin-like polypeptides, establishing machine learning as a robust descriptor-driven method for protein-based responsive polymers. More recently, Akar et al.^27^ reported a descriptor-based ML framework linking hydrophobicity metrics to thermoresponsive cloud points, followed by ANN-driven LCST prediction and temperature-responsive hydrogel swelling modeling in expanded data sets^28,29^ Recently, hybrid ML models incorporating physics-based descriptors were extended to pH-responsive hydrogel swelling and stress evolution, signaling the leap toward predictive, multistimulus polymer design.^30^ In 2019, Yoshida et al.^31^ first introduced a proof-of-concept study to illustrate a promising new example of polymer informatics while also raising many challenges that must be addressed before ML can be widely used. After nearly a century of development, polymer science has established a mature theoretical framework for understanding and predicting macromolecular behavior, as reflected in classical structure–property–performance relationships.^32^ The cause–process–structure–property–performance (CPSPPr) paradigm has long connected molecular features to macroscopic properties across length and time scales.^33,34^ Early property prediction relied on empirical inference and additive group contributions, while later studies revealed the importance of nonlinear and synergistic effects in structure–property correlations.^35^ In the current era of artificial intelligence, machine learning extends these foundations by enabling data-driven modeling and inverse design of polymers, complementing rather than replacing established polymer theory.^36^ Existing research^37–41^ shows that artificial intelligence and machine learning can accelerate polymer property prediction, composite design, and biomedical delivery by optimizing structures, additive manufacturing, and therapeutic kinetics. The amount of data on polymer materials is currently growing due to the quick advancement of data storage and experimental characterization technologies, particularly in the big data era. As a result, ML has also been extensively used in the development of polymer materials. Lu et al.^42^ reported the machine learning and polymer-related research published between 2012 and 2021. In this review we outline the development of stimuli-responsive polymer materials since 1995 and more precisely and specifically highlights their use in the field of AI/ML reported between 2018 and 2025. To cover the most current advancements in AI/ML-assisted polymer research, the time frame was limited to 2018–2025, which demonstrates an encouraging rise in the number of papers published throughout the course of the year. Following the first search, the articles that were gathered were filtered according to how relevant they were to AI and machine learning applications in polymer science, especially in fields like materials discovery, synthesis optimization, polymer design, and property prediction. The chosen papers were then divided into groups based on their main areas of interest, such as stimuli-responsive polymer systems, AI-guided polymer synthesis, data-driven materials discovery, and machine learning models for property prediction. Here, we pay special attention to supervised techniques and other data-driven approaches that work well for creating stimuli-responsive polymers. This review summarizes some of the most current studies in data-driven polymer research and attempts to offer complementary viewpoints on synthetic polymer design that have not been discussed in other review papers. A detailed roadmap for AI-driven polymer design and its applications is presented in this article. AI/ML-enabled techniques for creating stimuli-responsive materials come next, after the role of AI and machine learning in polymer science. The design of pH-, temperature-, and redox-responsive polymers, as well as AI-guided control in reversible deactivation radical polymerization (RDRP), are then the main topics of study. The use of machine learning techniques for polymer optimization and real-world applications, as well as the ML-driven design of amino acid–based polymers, are highlighted in later parts.^43^ Predictive models for performance evaluation, mechanism-aware and physics-informed learning methodologies, and the significance of AI in assessing toxicity, biocompatibility, and regulatory considerations for safe material applications are all included in this study.

It will be critical for chemists, data scientists, engineers, biologists, and regulators to collaborate across disciplinary boundaries. By taking these actions, AI/ML-guided stimuli-responsive smart polymers can progress from promising academic research to dependable, safe, and adjustable materials that tackle pressing issues in biotechnology, medicine, and other fields. Therefore, precision chemistry and intelligent chemistry are tightly related to smart responsive materials. AI/ML contributes to the generation of precise and accurate chemical data, which can subsequently be utilized to construct more intelligent chemical models, allowing for richer representations of complex chemical environments.

## 2 Designing pH-, Temperature-, and Redox-Responsive Polymers Using Artificial Intelligence

AI/ML enable codesign of pH,^44,45^ temperature,^29^ and redox-sensitive systems by learning nonlinear structure–property relationships, predicting stimulus thresholds, and optimizing compositions and architectures in high-dimensional spaces beyond intuition. Data-driven models have already been used to predict pH- and temperature-responsive behavior, reduce experimental iterations, and speed up the development of dual- and multistimulus-responsive platforms for targeted delivery and soft matter applications. According to precision chemistry’s vision, combining AI-guided molecular design, autonomous or semiautonomous synthesis, and in situ characterization will result in a new generation of smart polymers with quantitatively engineered pH, thermal, and redox responses for specific biological or technological applications. Nowadays, the AI/ML field is exploring the stimuli-responsive era in terms of responsive materials (Figure 1). pH- and temperature-responsive poly­(*N*-isopropylacrylamide-*co*-acrylic acid) (NIPAAm-*co*-AAc)-PEG IPN hydrogel was used for AI in modeling the doxorubicin release behavior. Prediction of the deswelling behaviors of pH- and temperature-responsive poly­(NIPAAm-*co*-AAc) IPN hydrogel by AI techniques. According to Yüceer et al.,^46^ AI—more especially, artificial neural networks (ANN)—were used to model the release of doxorubicin from pH and temperature-responsive poly­(NIPAAm-*co*-AAc)-PEG IPN hydrogels. Their findings demonstrated that the ANN model outperformed conventional regression techniques in accurately predicting drug release profiles based on crucial formulation and environmental characteristics, by facilitating quick and precise investigation of release behaviors under different pH and temperature. This data-driven method simplifies the optimization of smart polymeric drug delivery systems. The mechanical properties of IPN hydrogel that are dependent on temperature and pH were also described by this same group. It was discovered that the swelling capacity had an inverse relationship with the IPN hydrogels’ compressive strength. The least-squares support vector machine model and ANN model were utilized to create the model for the deswelling behaviors of IPN hydrogel. The ANN model’s predictions and the observed data have a very strong connection. The findings showed that the ANN model could correctly forecast the intricate deswelling behavior of IPN hydrogels that respond to temperature and pH.

**Figure 1:**
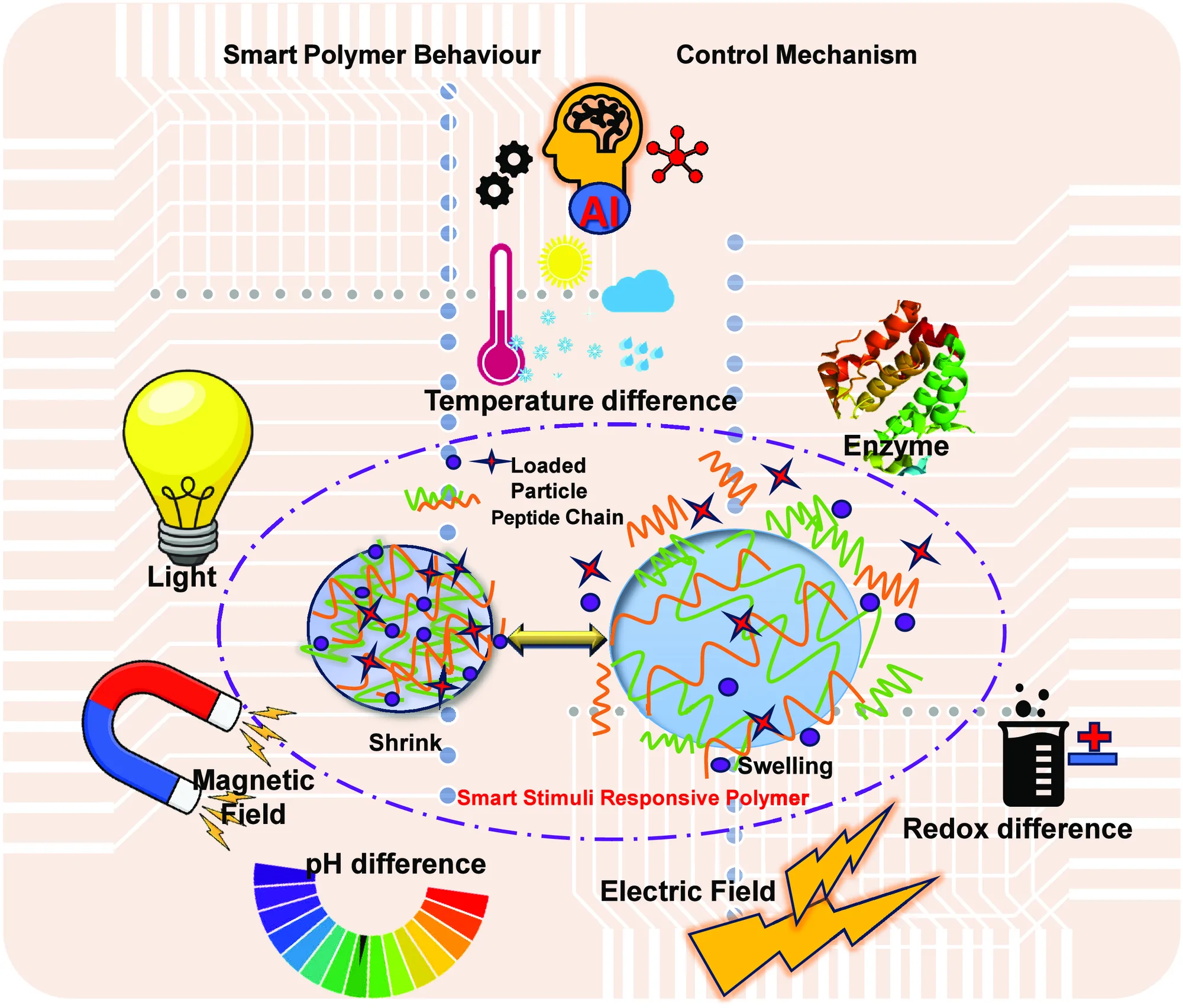
Artificial intelligence (AI) and machine learning (ML)–driven design, prediction, and control of stimuli-responsive polymer systems.

“Temperature responsive polymers”, also referred to as “temperature-dependent polymers”, are materials whose properties alter in reaction to temperature variations. Because those polymers may modify their physicochemical characteristics in response to temperature changes, they are employed in a wide range of applications, including drug delivery vehicles, sensors, and smart materials. The solvent–polymer H-bonding interaction controls this reversible transition, which allows the polymer solution to return to its original state as it cools. Examples of polymers exhibiting Lower critical solution temperature (LCST) are poly­(*N*-isopropylacrylamide) (PNIPAM),^47^ polyacrylamides (PAM),^48^ polyvinylethers (PVEs),^49^ polyoxazolines (POx),^50,51^ and poly­(oligoethylenenoxide)­methacrylates (POEGMA).^52,53^ It is worth noting that some temperature responsive also display an upper critical solution temperature (UCST)^54,55^ The deswelling behaviors of pH- and temperature-responsive poly­(*N*-isopropylacrylamide-*co*-acrylic acid) poly­(NIPAAm-*co*-AAc) IPN hydrogels were predicted by Boztepe et al.^46^ using artificial intelligence approaches, particularly ANN. Their results showed that the intricate, nonlinear relationship between environmental triggers and the hydrogel’s physical reaction may be accurately captured by ANN models. The accurate prediction and control of hydrogel swelling and deswelling dynamics made possible by this AI-driven modeling approach are essential for creating sophisticated, intelligent drug delivery devices. The development of next-generation smart biomedical materials is accelerated by this synergy between pH-responsive polymers and AI/ML.^56^ Redox stimulation is an electrochemical procedure that modifies the oxidation state of a redox-responsive group. This kind of stimulation is really more common in inorganic chemistry, especially for the transition metals. The polymer chain’s redox-responsive group enables the synthesis of redox-responsive polymers. To synthesized this kind of polymer, a variety of functions have been added to the polymer. Examples include dithienylethenes,^57^ ferrocene,^58^ and disulfides.^59^ Tetramethylpiperidine-1-oxyl (TEMPO) derivatives, which are paramagnetic organic molecules, are a redox-responsive moiety that is stable and has a long reversibility. ML is increasingly central to accelerated discovery and optimization of redox-responsive polymer systems. Flox et al.^60^ reported a study where supervised models, active learning, and physics-informed surrogate modeling were combined to screen redox couples, predict performance metrics, and guide experiments for redox flow batteries, achieving faster optimization of capacity and cycling stability than trial-and-error approaches. Kuenneth et al.^61^ reported an AI-driven framework that fuses large battery data sets with meta-learning to predict voltage, specific capacity, and electronic conductivity of polymer electrodes, enabling inverse design and prioritization of promising organic electrode candidates. Together these works demonstrate that ML workflows — DFT-enhanced feature engineering, uncertainty-aware acquisition, and multiobjective optimization — can reduce experimental load and accelerate scale-up. The studies recommend integrating explainable models and automated synthesis-characterization loops to turn ML candidates into deployable next-generation organic batteries. Open data and community benchmarks are urged to improve reproducibility.

## 3 AI/ML-Enabled Methods for Developing Materials That Respond to Inputs

AI/ML advancements have greatly improved the development of responsive materials Designing active composites that may undergo predictable shape transitions remains both desirable and problematic. AI/ML are becoming increasingly popular due to their capacity to provide speedy, cost-effective, and parallelised solutions to such complex design difficulties.^62,63^ In Figure 2, we described how the research area has subsequently progressed, including recurrent neural networks, support vector machines, and a greater emphasis on data-driven approaches.^64,65^ Recent developments in computer hardware have enabled AI/ML techniques to solve multiple problem domains, such as picture identification, music and video streaming, and various optimization tasks.^66^ Evolutionary algorithms (EAs) are machine learning methods that solve multiobjective optimization and combinatorial issues. Inspired by natural evolution, EAs apply the “survival of the fittest” idea to a computational framework, allowing solution populations to compete until the best one emerges.^67–69^ EAs have been used to train AI for optimizing neural networks, composite design, and other engineering domains.^66^

**Figure 2:**
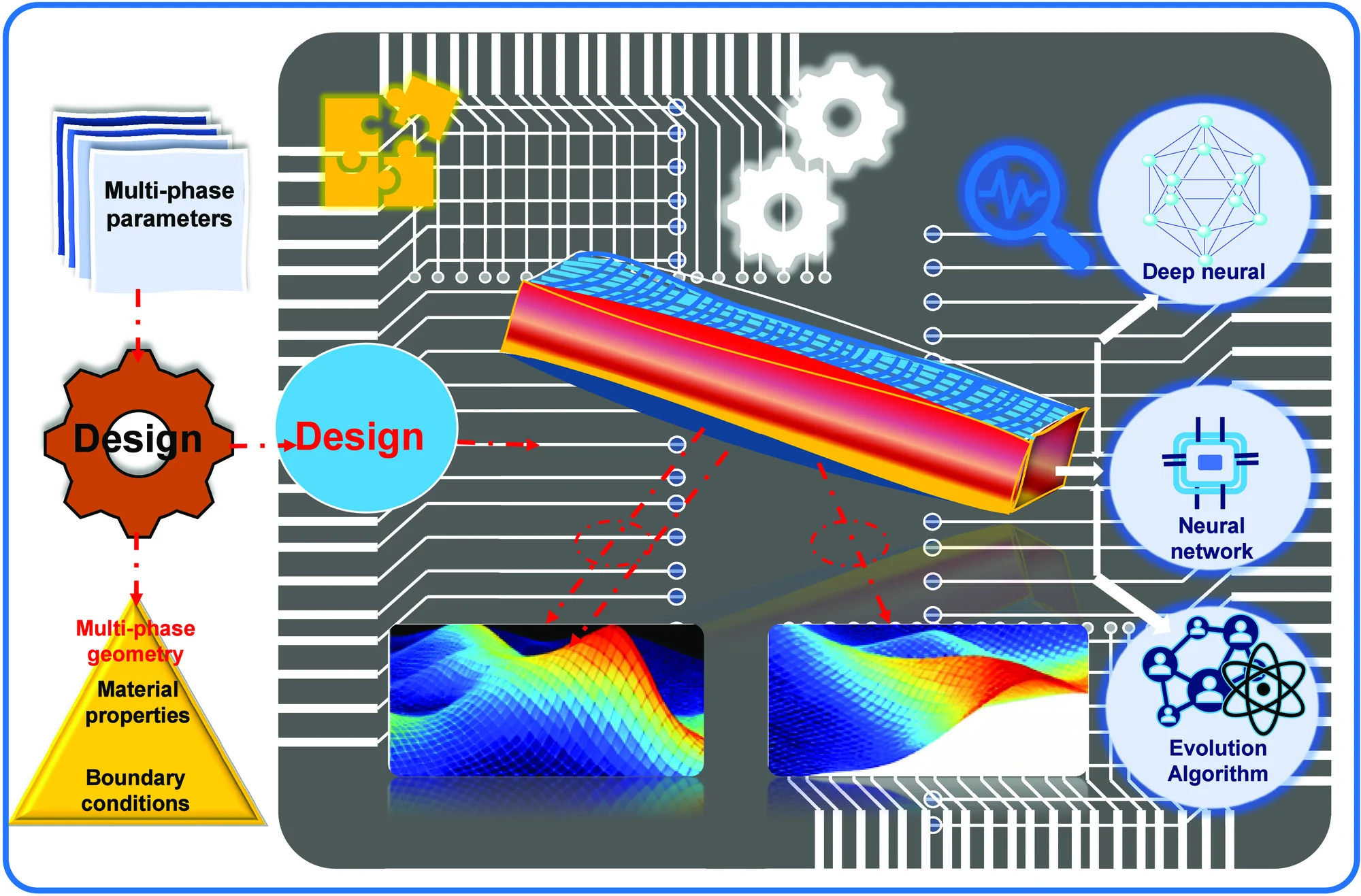
AI/ML-enabled multiphase design and optimization framework for polymer and soft-material systems.

### 3.1 More Than Static Properties: Modeling Kinetics, Hysteresis, and Fatigue

In the past, machine learning was applied in polymer science to forecast static equilibrium parameters like the ultimate swelling ratio or compressive modulus. However, the main benefit of stimuli-responsive polymers is their ability to change dynamically. For practical use in soft robotics or medication distribution, the “smart” behavior is characterized not only by the end point but also by the kinetics of the reaction, the switching thresholds, and the ability to go back and forth over and over again.

### 3.2 Anticipating Switching Thresholds and Phase Transitions

For biomedical effectiveness, it is very important to have the transition points, like the Lower Critical Solution Temperature (LCST) or pH-switching thresholds, just right. Conventional theoretical models frequently encounter difficulties in forecasting these thresholds in intricate multicomponent copolymers because of nonideal mixing behaviors. Recent data-driven methodologies have proven that deep learning architectures may delineate nonlinear relationships between monomer sequences and phase transition temperatures.

For example, machine learning models have been used to separate the opposing effects of hydrophobic and hydrophilic parts on the coil-to-globule transition in poly­(*N*-isopropylacrylamide) (PNIPAM) systems. This makes it possible to build polymers with specific switching windows.

### 3.3 Hysteresis Loops and Response Kinetics

One of the most significant problems with designing smart polymers is the “responsiveness mismatch”, which happens when the material does not respond quickly enough to changes in the environment. This happens because of diffusion kinetics and polymer chain relaxation, which often leads to a lot of hysteresis, where the actuation path is different from the recovery path. Researchers are moving away from static feed-forward networks and toward sequence-based architectures to better understand how things change over time. Long Short-Term Memory (LSTM) networks are very useful in this case. Recent research on polymer matrix composites has shown that LSTMs can keep track of how material properties change over time. LSTMs have internal “memory” states that let them work with sequential data, which makes them better for modeling than regular regression. Path-dependence: Predicting the current state of a shape-memory polymer based on both its current temperature and its thermal history.

Viscoelastic relaxation: Simulating the time delay in hydrogel actuation during fast swelling and deswelling cycles, which makes it quite accurate in predicting where failures would happen.

### 3.4 Modeling of Fatigue and Degradation Based on Physics

For commercial viability, stimuli-responsive systems must withstand thousands of actuation cycles without mechanical breakdown. Models that are based only on data may not be able to predict failure modes that are not in their training data. Physics-informed neural networks (PINNs) provide a strong answer to this problem. PINNs ensure that predictions follow thermodynamic rules by putting governing equations like balancing laws, diffusion-reaction kinetics, or phase-field formulations directly into the loss function.

## 4 AI-Guided Precision Design and Control for Reversible Deactivation Radical Polymerization

Machine learning has so far been most concretely connected to ATRP and RAFT within the RDRP family, with only scattered or indirect ties to OMRP, ITP, cationic, and anionic polymerizations; thus, only ATRP and RAFT can currently be studied in a focused AI/ML formulation context. Because explicit AI/ML-driven formulation frameworks could not be identified for OMRP, ITP, cationic, or anionic polymerizations, these techniques are not included here, in line with the scope.^70^

### 4.1 Atom Transfer Radical Polymerization (ATRP): AI/ML Optimized Synthetic Pathway

AI/ML was utilized to (i) learn kinetic relationships, (ii) optimize reaction circumstances, and (iii) actively control ATRP in real time. A seminal work used deep reinforcement learning on a mechanistic ATRP simulator to establish dosing regimens for catalyst and initiator, resulting in targeted, even non-Gaussian molecular weight distributions and exhibiting AI-based control over chain statistics. Recent work incorporates machine learning into automated or semiautomated photo-ATRP platforms to predict reaction conditions (catalyst, ligand, light, concentration, solvent) for desired conversion and dispersity, reducing the need for physical experiments for optimization. ML-derived propagation rate models for radical polymerization constants feed into kinetic ATRP simulations, allowing for more precise in silico recipe design for specific molecular weights and structures. The activation–deactivation mechanism of atom transfer radical polymerization (ATRP) is derived from atom transfer radical addition (ATRA), a technology discovered in the 1960s that allows the addition of carbon–carbon double bonds to alkyl halides via transition metal catalysis. Krzysztof Matyjaszewski improved the mechanical and practical features of ATRP, establishing it as a versatile and controllable polymerization process.^71,72^ The significant discoveries include improved control over polymer design, increased utilization in aqueous and heterogeneous settings, and successful reduction of copper catalyst levels to the ppm range using ARGET and initiators for continuous activator regeneration (ICAR) ATRP. Figure 3 illustrates the pathway of ATRP in terms of AI ML formulation. In ATRP, Yaron et al.^73^ synthesized a revolutionary method to manipulate the form of polymer molecular weight distributions (MWDs). This approach takes advantage of recent improvements in ATRP numerical modeling, as well as simulation-based, model-free reinforcement learning (RL). An ATRP simulation environment is created, which allows an RL controller to inject chemical reagents into the reaction. The controller uses fully connected and convolutional neural network architectures to make decisions based on the condition of the ATRP system. Because the initial untrained controller generates extremely variable final MWDs, the RL algorithm investigates a wide search space. When trained with an actor-critic framework, the RL controller effectively finds and optimizes control strategies to produce a variety of target MWDs, including Gaussians with varied widths and complex forms like bimodal distributions. Even in the presence of simulated noise, the learnt control policies remain durable and transfer to similar but distinct ATRP reaction scenarios. Banin et al.^74^ reported artificial neural networks toward average characteristics targets in styrene Activators regenerated by electron transfer atom transfer radical polymerization (ARGET-ATRP). For the first time, ARGET-ATRP used artificial neural networks (ANNs) to synthesize polystyrene. In this study, deterministic modeling was used to create an ANN capable of predicting reaction conditions based on the average properties of the target polymer and vice versa. Zhao et al.^75^ have demonstrated a “plug-and-play” solution for simplified electrochemical atom transfer radical polymerization (e-ATRP), which was evaluated using machine learning. The polymerization of oligo­(ethylene glycol methyl ether acrylate)_480_ (OEGA_480_) in water is shown to be more efficiently optimized through Bayesian optimization driven by an active learning (AL) algorithm. The selected optimization output targets poly­(oligo­[ethylene glycol methyl ether acrylate]) (POEGA_480_) with a low dispersity (\*Đ*_*m*_ < 1.30), where the molecular weight distribution (*M*_w_/*M*_n_; dispersity; \*Đ*_*m*_) serves as the key performance parameter. Gormley et al.^76^ described a workflow for oxygen-tolerant photo-ATRP that uses automation to speed up polymer synthesis, produce high-throughput data, and use machine learning to optimize reactions. ML-assisted ATRP has been used to find and optimize functional ATRP-derived copolymers, such as TSCT emissive systems. Predictive models connect monomer/initiator structure to optical characteristics, directing polymer composition and degree of polymerization as set by ATRP.

**Figure 3:**
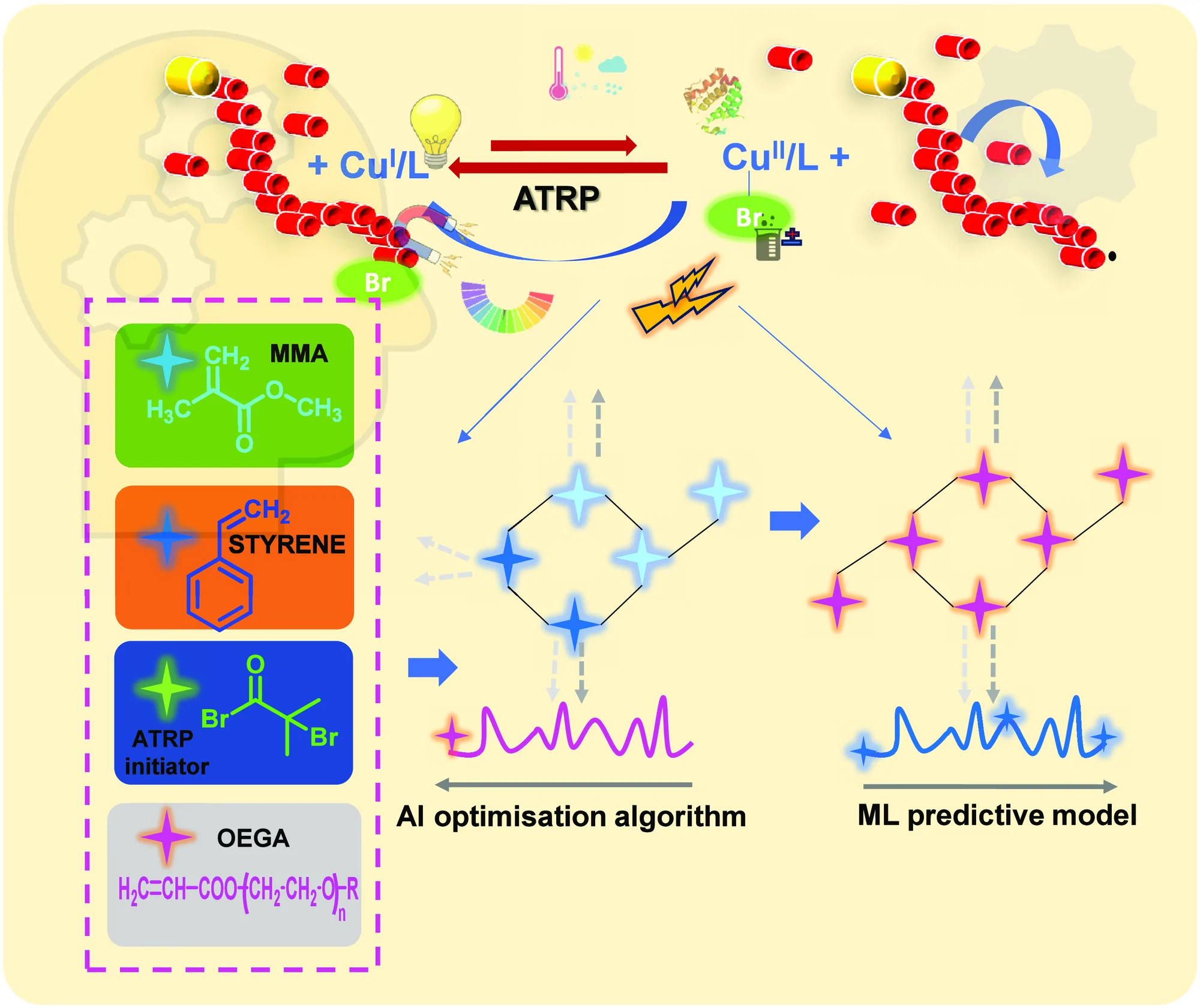
AI/ML-assisted optimization and prediction of ATRP-controlled radical polymer synthesis.

### 4.2 Reversible Addition–Fragmentation Chain Transfer Polymerization (RAFT): AI/ML Optimized Synthetic Pathway

AI/ML has been integrated into RAFT’s batch and continuous flow platforms to optimize polymerizations autonomously. A flow reactor with in-line NMR and SEC was combined with machine-learning-guided self-optimization to tune RAFT conditions (temperature, residence time, monomer:CTA: initiator ratios) for targeted conversion, molar mass, and dispersity. This demonstrates closed-loop RAFT process optimization. Predictive ML models for propagation rate coefficients and structure–reactivity relationships in radical copolymerization are being integrated into RAFT kinetic simulations. This allows for data-driven design of RAFT recipes and architectures with targeted chain length distributions and block sequences.^77^

Reversible addition–fragmentation chain transfer (RAFT) is a popular approach for creating polymers with precise topologies (Figure 4). This living radical polymerization process is guided by the RAFT agent, promoting the production of polymer chains with high-fidelity end groups.^78^ Sustainable, high-performance materials can be discovered efficiently and in time to meet societal demands only through the integration of artificial intelligence and machine learning. According to Wilding et al.^79^ a new mechanistic model for reversible addition–fragmentation chain transfer polymerization (RAFT) enables the training of a Bayesian optimization algorithm utilizing in silico processes. Karayilan et al.^80^ used a Chemspeed robotic platform for batch, incremental, and continuous monomer addition under inert circumstances to describe an automated RAFT copolymerization process. Time-resolved ^1^H NMR spectroscopy was used to study the reaction kinetics of copolymerising oligo (ethylene glycol) acrylate with fluorescein-o-acrylate in various solvents. Lima et al.^81^ validated a sequential Bayesian technique for discriminating models in RAFT polymerization. The validity of RAFT models, which are complete nonlinear mechanistic models taken from literature, is still debated. To select the most informative measured outputs for model discrimination, sensitivity studies of the simulated models were first carried out. Using artificial intelligence and machine learning in polymer development can help meet the demand for high-performance, sustainable materials. Warren et al.^82^ demonstrated that various RAFT polymerization formulations can self-optimize in a computationally controlled flow reactor. This is the first example of closed-loop (user-free) optimization for multiple targets in polymer synthesis, enabling autonomous identification of optimal reaction conditions for desired polymer features. The synthesis platform used a computer-controlled flow reactor, online benchtop NMR, and in-line GPC. The Thompson sampling efficient multi-objective optimization (TSEMO) algorithm was used to optimize the RAFT polymerization of *tert*-butyl acrylamide (tBuAm), *n*-butyl acrylate (BuA), and methyl methacrylate (MMA), analyzing trade-offs between molar mass dispersity and monomer conversion without user intervention.

**Figure 4:**
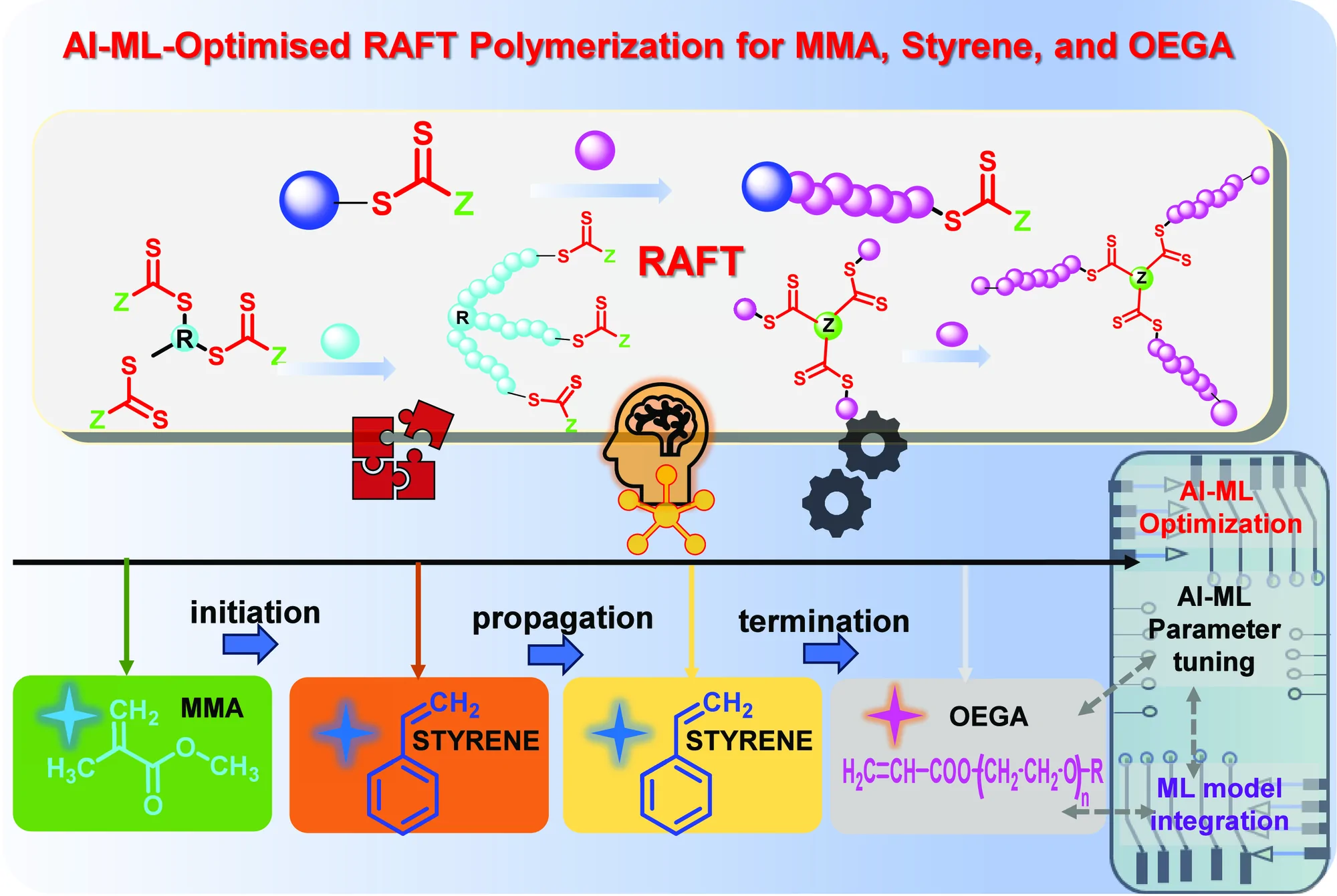
AI–ML-optimized RAFT polymerization for precision synthesis of MMA-, styrene-, and OEGA-based polymers.

### 4.3 Nitroxide-Mediated Polymerization (NMP): AI/ML Optimized Synthetic Pathway

A (macro) alkoxyamine is the main species in nitroxide-mediated polymerization (NMP), existing in reversible equilibrium between the active and dormant phases.^83^ NMP provides a simpler and more ecologically friendly method than other controlled radical polymerization procedures since it does not require metal catalysts or bimolecular exchange processes. Pyrrolidone-enriched copolymers were synthesized by Savelyeva et al.^84^ Recently, an alkoxyamine molecule was developed that, when added to polyurethane, gave it the capacity to mend itself at room temperature. This method successfully designed epoxy-based polymer systems and polyurethane copolymers using heat stimuli.^56,85^ Gao et al.^86^ described an active learning strategy employing a cluster-maximum model change (CMMC) approach to map the relationship between polymerization conditions and key parameters, including *M*_*n*_, PDI, skewness, and kurtosis. They demonstrated that directing kinetic Monte Carlo (KMC) simulations through active learning can substantially reduce computational effort. In their nitroxide-mediated polymerization (NMP) model of styrene, alkylnitroxide acted as the initiator. The study aimed to address a two-stage optimization problem using active learning: (1) Optimal initialization strategy — given *N* unlabeled samples, identifying the most informative initial samples for annotation to construct an improved first prediction model (*f*_0_); and (2) Optimal relabeling strategy — utilizing the initial model (*f*_0_) to select the most valuable samples for parameter refinement and to build the final predictive model at minimal computational cost.

## 5 AI/ML-Driven Design of Stimuli-Responsive Amino Acid Polymers

Amino acid-based stimuli-responsive polymers (Figure 5) combine the sequence specificity and biocompatibility of peptide building blocks with traditional polymer design to produce materials whose assembly and function may be controlled by environmental cues (pH, temperature, redox, ions, light, enzymes). De et al.^87^ reported that natural and synthetic amino-acid motifs can be incorporated as side chains, backbone repeats, or end groups to impart reversible conformational switching, amphiphilicity changes, or cleavage-triggered disassembly, enabling applications ranging from controlled drug release to adaptive hydrogels and biosensors. Design tactics rely on weak, reversible interactions (hydrogen bonding, π–π stacking, electrostatics, and hydrophobic collapse) that can be adjusted through residue selection, sequence patterning, and chain architecture. For example, pH-responsive behavior is frequently created by incorporating ionizable residues (Asp, Glu, Lys, and His) so that protonation changes affect solubility and self-assembly, whereas thermoresponsive motifs such as elastin-like repeats allow for abrupt, temperature-dependent phase transitions. These modular design features make amino-acid-derived polymers especially appealing in applications where biocompatibility and degradation profile are crucial.^88^ Despite these benefits, the compositional and sequence space of peptide-inspired polymers is enormous, and typical trial-and-error synthesis and characterization slow down rapid optimization. Artificial intelligence (AI) and machine learning (ML) technologies have begun to speed up formulation and discovery by mapping structure–property correlations from diverse experimental and simulated data sets. Supervised learning (random forests, gradient boosting, neural networks) has been used to predict macroscopic outputs from molecular descriptors and processing conditions, whereas unsupervised and active-learning strategies prioritise experiments to maximize model information.^89^ Practical AI-assisted workflows for amino-acid–based stimuli-responsive systems combine curated data sets (composition, sequence motifs, molecular weight, solvent, pH, temperature), feature engineering (residue hydrophobicity, charge distribution, predicted secondary structure), and multitask models that predict switching temperature, mechanical modulus, and degradation rate. Combining ML predictions with high-throughput synthesis (solid-phase peptide assembly, controlled polymerization) and rapid screening (microplate turbidity, automated rheology) allows for closed-loop optimization: models propose candidate sequences/formulations, automated synthesis produces them, characterization feeds results back to the model, and the cycle quickly converges to target performance.^90^ Key problems remain: few public data sets that are standardized for peptide–polymer formulations, model interpretability for mechanistic insight, and transferability between chemistries and scales. To close these gaps, the community must work together to share curated experimental data, adopt physics-aware ML (hybrid models, PINNs) that incorporate thermodynamic/kinetic constraints, and conduct validation campaigns that link ML-predicted formulations to application-relevant end points (biocompatibility, long-term stability). When amino-acid motifs’ molecular precision and AI/ML’s predictive power combine, they promise to revolutionize the rational design of stimuli-responsive polymers for biomedical and precision manufacturing applications.

**Figure 5:**
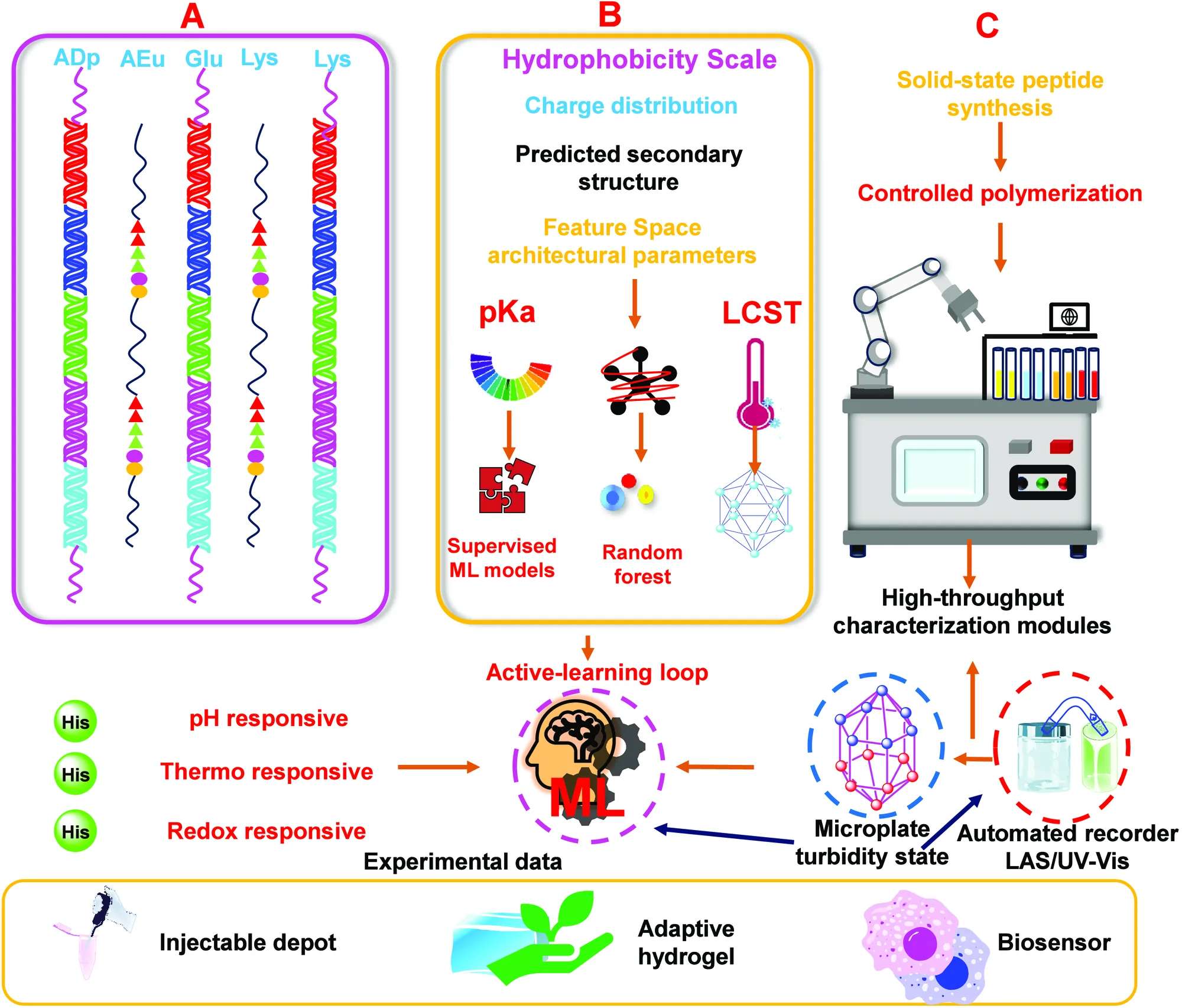
Active-learning-guided design and synthesis of peptide–polymer hybrids for multistimuli-responsive biomaterials. (A) Amino acid–based sequence with charge distribution (p*K*_a_ scale) and anticipated structures. (B) Maps p*K*_a_ to LCST in architectural feature space via supervised ML (random forest) and an active learning loop for structural optimization. (C) High-throughput controlled polymerization for robotics, characterization (LA-UV–vis), and applications (injectable depot, biosensor).

## 6 New Approach to Machine Learning in Polymer Science

ML methods are revolutionizing polymer research by replacing trial-and-error experimentation with data-driven discovery, enabling quick mapping from structure and processing conditions to characteristics and performance, as illustrated in Figure 6. By merging autonomous synthesis, property prediction, and generative design, ML provides the essential tools for a systematic, predictive, and genuinely innovative approach to polymer research.^91^ Summary of recent machine-learning-assisted polymerization methodologies, learning tasks, and model trade-offs is provided in Table 1. The development of AI is based on data, wherein the physical world’s materials and substances are separated into data and purpose-oriented models. ML models trained on near-infrared, Raman, and UV–vis spectrum data may effectively monitor polymer processing, operation, and deterioration. Research has examined hot-melt extrusion^92^ polymer electrolyte membrane fuel cells (PEMFCs)^93^ and the presence of faults in cell stacks at early phases.^94^ An online sequential classifier using transfer learning was developed to detect Mura defects in organic and polymer light-emitting diode displays.^95^ The use of ML and deep learning to process vast amounts of data and information in the pursuit of knowledge and wisdom (DIKW pyramid) has grown rapidly. AlphaGO for Go, AlphaFold for protein folding, and ChatGPT for natural language processing are just a few of the achievements that came from it. AI/ML approaches are rapidly shifting polymer research from empirical screening to data-driven design. Malashin et al.^96^ reported that ML workflows can compress multivariate structure–property landscapes into tractable surrogate models. This enables prioritisation of candidates for synthesis and testing. Further, they have showcased the usefulness of ensemble tree methods and boosting algorithms for predicting composite and polymer blend properties. As shown in Table 1, the successful use of ML algorithms in polymerization studies is dependent on properly aligning the model’s architectural strengths with specific task needs and data constraints. For predicting complex, nonlinear outputs like molecular weight distributions or power conversion efficiencies, ensemble tree methods (e.g., Random Forest, Gradient Boosting, XGBoost) frequently succeed. The cause is, these methods provide high accuracy while maintaining critical posthoc interpretability (such as SHAP values) to analyze feature importance. Conversely, when the inputs are raw molecular structures rather than engineered descriptors, graph-based models (like graph attention networks or MIMOGAN) outperform traditional regressors by natively capturing spatial topology to predict properties like refractive indices or reactivity ratios. Furthermore, researchers successfully navigate the trade-offs of “small data” bottlenecks by employing transfer learning—such as fine-tuning a pretrained Deep Neural Network on limited batch data—or by coupling standard supervised regression models with genetic algorithms to efficiently inverse-design materials across high-dimensional parameter spaces. Datta et al.^97^ highlighted that data-driven methods are particularly valuable for stimuli-responsive amino-acid polymers because responses (pH, temperature, redox, light) often depend on subtle, nonlinear interactions between sequence, side-chain chemistry, and supramolecular assembly. The FDA SafetAI initiative was cited as an example of regulatory interest in ML-based prediction of safety end points, illustrating translational potential beyond materials optimization.^98^ Data-driven and physics-based innovations can yield actionable insights and design principles by leveraging the composition-process-structure–property-performance relationship (CPSPPr)^22,34^ framework, which provides a unified description of material behavior throughout the polymer life cycle. Polymer materials have the fastest rate of development among the four primary types of materials—metals, inorganic nonmetals, organic polymers, and composites—due to their adjustable chemistry, hierarchical topologies, and process sensitivity. As a result, CPSPPr serves as a particularly useful framework for polymers, allowing for systematic linkages between molecular composition, processing history, changing structure, emergent features, and application-level performance, including stimuli-responsive capabilities.

**Figure 6:**
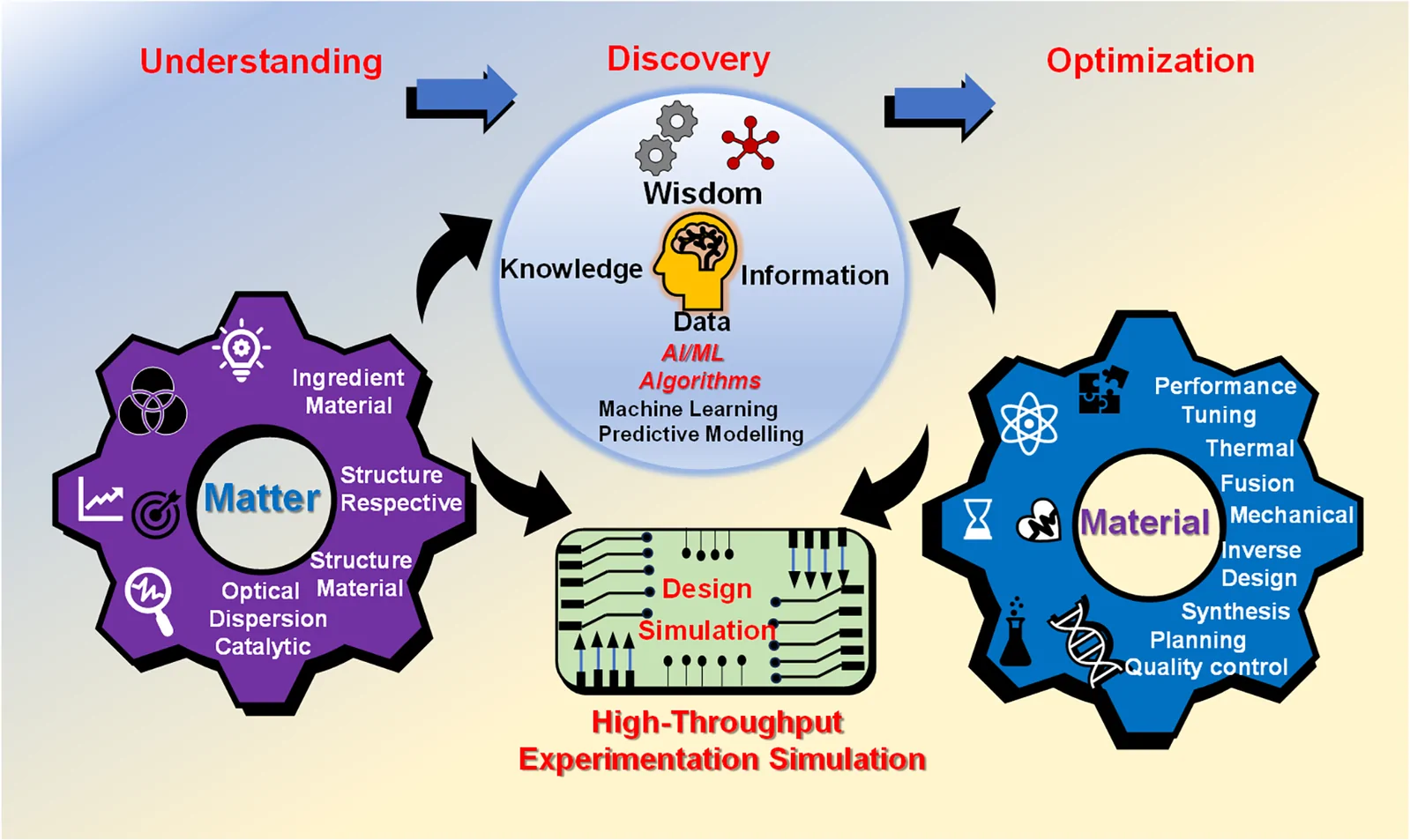
Understanding, discovering, and optimization of materials using AI ML. Reproduced with permission.^99^ Xie et al. Copyright 2025, John Wiley and Sons, Creative Commons CC BY license, open access.

**Table 1:** Examples of Machine-Learning-Assisted Polymerization Studies That Summarize Polymerization Methodologies, Important Methodological Advances, Learning Tasks, and the ML Algorithms Used

Reference	Polymers/Polymerization Technique	Key Novelty	Task	ML Algorithm(s)
Tan et al.^100^	1. High-throughput computer-controlled polymerization; molecular weight distribution control	Transfer learning for prediction of full molecular weight distributions	1. Regression of styrene monomer conversion from 3D input space (styrene wt fraction, styrene: AIBN molar ratio, residence time).	5. Random Forest regressors for conversion prediction.
			2. High-dimensional regression for predicting full MWD weight-fraction vectors.	6. Gradient Boosting/XGBoost-style tree ensembles as part of model comparison for conversion and/or MWD prediction.
			3. Transfer learning from flow to batch: fine-tuning a pretrained DNN on smaller batch MWD data set.	7. Deep Neural Networks (fully connected DNNs) for predicting full MWDs, including smoothed-output architectures.
			4. Model interpretation/feature importance analysis for conversion and MWD summary metrics.	8. SHAP (SHapley Additive exPlanations) for posthoc interpretability of tree-based models and DNNs.
Nguyen et al.^101^	Copolymerization systems; polymer reaction engineering.	Predict monomer reactivity ratios directly from molecular structure using a graph-based ML model.	Supervised prediction of copolymer reactivity ratios r1, r2 from structures of both monomers.	Multi-Input-Multi-Output Graph Attention Network (MIMO-GAN).
Lo et al.^102^	Polymer structure data sets (no synthesis focus).	Proposes “iterative rearrangement” as a data-augmentation scheme that generates multiple equivalent polymer representations (e.g., rearranged SMILES/SELFIES, fragments) while preserving connectivity and sequence information.	Supervised polymer property prediction on three data sets (e.g., bandgap, *T*_g_, solubility) using different molecular representations, with and without augmentation.	Standard ML regressors/classifiers commonly used in polymer informatics (e.g., tree-based models and neural networks) trained on baseline vs augmented representations (exact model names are detailed in the Supporting Information).
Ihara et al.^103^	Polymer–solvent systems analyzed in Hansen solubility space.	Discovery of a new Hansen-space axis indicating spherical precipitation	1. Classify solvent pairs (good/poor) as giving spherical vs nonspherical polymer precipitates based on solvent physical properties and Hansen parameters.	3. Random Forest to rank feature importance and select key solvent descriptors or principal components.
			2. Train SVM classifiers on selected features (original descriptors and PCA-derived PCs) to learn decision boundaries between spherical and nonspherical precipitation.	4. Support Vector Machine (SVM) for binary classification of spherical vs nonspherical precipitation in the reduced feature space (including PC4).
Song et al.^104^	Poly(aryl ether)s; Polymer	Genome-driven design of high-*T*_g_ polymers	Supervised prediction of glass transition temperature *T*_g_ of poly(aryl ether)s from monomer structures and feed ratios.	Machine learning regression models (ensemble and neural-network type) trained on polymer-genome features to predict *T*_g_ and rank favorable/unfavorable.
Lu et al.^105^	Particulate-filled polymer composites.	Data-driven prediction of interfacial thermal resistance	Predict unknown interfacial thermal resistances and effective thermal conductivity of particulate-filled polymer composites from limited experimental TC data plus detailed microstructural descriptors.	Supervised ML regression models (compared in the SI) to fit effective TC vs structural/ITR parameters, coupled with a genetic algorithm to inverse-identify ITR values consistent with experiments.
Cheng et al.^106^	Polymer–metal tribological systems.	Use of friction noise signals for property prediction	Supervised regression of friction coefficient for four polymers–seven metals under multiple temperatures and load/speed conditions, using extracted friction-noise features.	Gradient boosting ensemble models: XGBoost, LightGBM, and CatBoost compared for friction-coefficient prediction from noise features.
Xu et al.^107^	Thermoplastic vulcanizates (TPVs); dynamic vulcanization.	Processing–property mapping via ML	Predict tensile strength and elongation at break of PLA/ENR and PP/EPDM TPVs from material features (rubber type/content, vulcanizing system, fillers) and processing conditions (speed, temperature, etc.).	GA-BP model: Backpropagation neural network whose weights and topology are optimized by a genetic algorithm, plus partial-dependence analysis for factor effects.
Mishra et al.^108^	Computationally generated amorphous polymers.	High-throughput RI prediction integrating.	Predict RI of amorphous polymers from structural/compositional descriptors built from computationally synthesized polymer structures.	Graph Attention Neural Network (GAT-type) model for supervised prediction of polymer RI from graph-based polymer representations.
Agarwala et al.^109^	Semiconducting polymer melts.	ML identification of strong electronic contacts	Predict electronic coupling strength between neighboring P3HT monomers from a compact set of geometric descriptors, instead of performing explicit quantum-chemical calculations for every pair.	Supervised ML model with automated feature selection (sparse/regularized model on geometric features; details in full text) trained to regress electronic coupling from local structural descriptors.
Yang et al.^110^	MXene/PPy/HEC polymer composites; in situ polymerization.	ML-enabled handwriting recognition using flexible strain sensors	Classify handwritten characters from strain-sensor time-series signals recorded during writing.	Standard supervised classifiers (e.g., SVM/NN-type models; exact model list given in Supporting Information) trained on sensor signal features for character recognition.
Cicek et al.^111^	Biopolymer iontronic sensors.	Ultrasensitive tactile sensing for object recognition	Object recognition from tactile signals	Conventional supervised classifiers (Naïve Bayes, SVM, k-NN, Random Forest, Discriminant Analysis, Decision Tree) trained on sensor features; best models reach ≈90% accuracy.
Zhao et al.^112^	Binary organic solar cells (Y6-based polymers).	ML-based PCE prediction and material screening	Supervised prediction of power conversion efficiency for small-molecule-donor/Y6 and polymer-donor/Y6 devices from computed donor molecular descriptors.	Six algorithms: Gradient Boosting Regression Tree (GBRT), Random Forest, XGBoost, AdaBoost, Support Vector Regression (SVR), and Multiple Linear Regression (MLR); tree-based methods, especially GBRT, performed the best.

## 7 AI and ML Play an Important Role in Designing Stimuli-Responsive Materials

The use of artificial intelligence in materials science has opened a new era of data-driven innovation and complements conventional research methods (Figure 7). It can also predict how they will act in certain situations, which makes it possible to make materials that really respond to their environment. This AI-driven method speeds up the search for new polymer compositions that are more durable, flexible, and resistant to the environment. This helps both high-performance and biodegradable materials get better.^96^ AI-based solutions are becoming more and more important since modern polymer systems are becoming more complicated and must meet high standards for performance, safety, and sustainability. AI-guided discovery is also making breakthroughs in health and environmental uses. For instance, researchers can now create and change biomaterials according to the needs of a certain disease or even a patient’s own biological environment.^113^ Recent studies have described data-driven advancements in quantitative presentation of conformations, properties, and Quantitative Structure−Property Relationships (QSPRs), leading to the development of new techniques and algorithms using expert-annotated data sets.^114,115^ ML’s capacity to recognize chemical structures and phase domains improves property prediction and segmentation, leading to faster and more accurate characterization procedures.^116^ A cutting-edge example comes from a research team at Cornell that built a hybrid quantum-classical AI framework for making new peptides, which are chains of amino acids that are used to clean up microplastics. This method combines generative AI models with quantum computing to quickly look at thousands of amino acid sequences at the same time. This speeds up the search for peptides that bind strongly to plastics like polyethylene terephthalate (PET), polyethylene, and polypropylene. This is a big step toward AI-assisted and environmentally friendly solutions. AI gives us more profound insights and faster innovation by finding high-dimensional, nonlinear correlations between polymer formulation and performance that traditional experimental methods typically miss. Consequently, researchers can more efficiently formulate next-generation polymers that address the changing requirements of modern technologies and environmental challenges.

**Figure 7:**
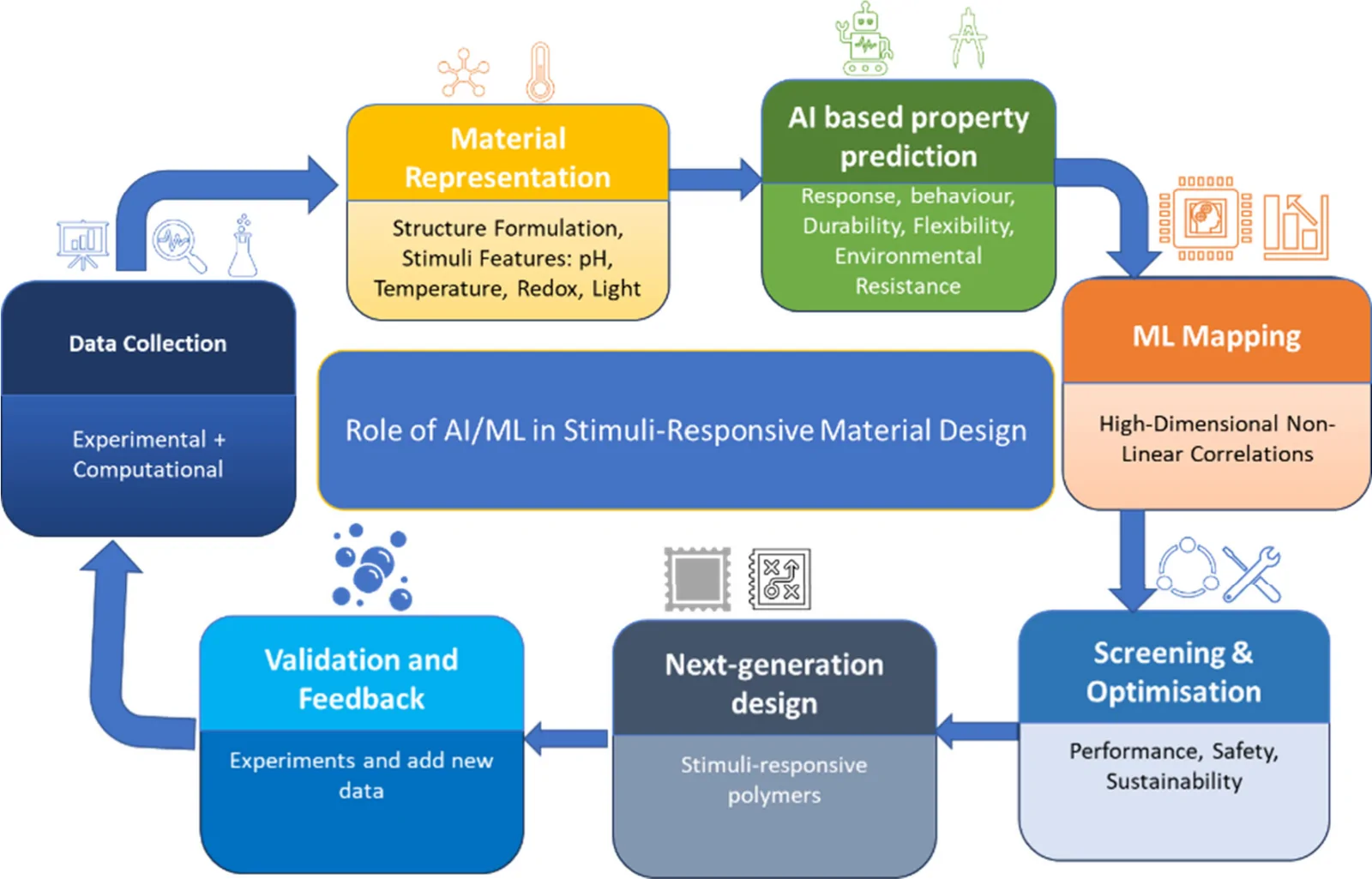
End-to-end AI/ML workflow for the rational design of stimuli-responsive materials

## 8 Machine Learning Algorithms for Polymer Optimization

There are a lot of ML algorithms being used in polymer science right now. Each algorithm is suitable for certain problems that come up when designing materials and improving processes. Table 2 summarizes key the Machine Learning Algorithms in polymer properties.

- **Regression Models:** Algorithms, e.g. Gaussian process regression (GPR), have been employed with significant accuracy to predict important features like the tensile strength of polymer/carbon nanotube nanocomposites. Furthermore, Random forest regressors have also been employed with greater precision to forecast how long materials will naturally age, with a high R^2^ value of 0.92.^96^
- **Boosting Methods:** This group of algorithms, which includes AdaBoost, Gradient Boosting, XGBoost, CatBoost, and LightGBM, has become a powerful way to solve the complex, high-dimensional problems that are common in polymer science. These models have effectively been employed to predict the rheological characteristics and thermal deflection temperatures of composites.^117^
- **Deep Learning:** Ivan et al.^96^ had concluded that Artificial neural networks (ANNs) and convolutional neural networks (CNNs) are commonly used for predictive modeling and finding defects in textiles made of polymer. Furthermore, these models can assist in speeding up any polymer-based research work by significantly reducing the need for long cycles of trial and error.
- **Optimization Algorithms:** Algorithms like Ant colony optimization (ACO) have been used to improve manufacturing processes, like identifying the best dye recipes for textiles, in addition to making predictions. Bayesian optimization can also be used to find the best circumstances for a reaction on a global scale, as shown by the creation of conductive e-textile prototypes.^96^

**Table 2:** Key Machine Learning Algorithms and Their Application in Polymer Properties

Algorithm Class	Specific Example	Polymer Property Target	Insight/Advantage
Regression Models	Gaussian Process Regression (GPR)	Tensile Strength, Natural Aging Times	Bayesian, nonparametric method ideal for data-sparse, high-dimensional R&D, providing uncertainty quantification.
Boosting Methods	XGBoost, Gradient Boosting	Rheological Properties, Heat Deflection Temperature	Excels in capturing complex, nonlinear relationships inherent in composite materials, suitable for efficient processing of mixed data types.
Deep Learning	ANNs, CNNs	Density, Viscosity, Defect Detection in Textiles	Capable of learning multiscale structural representations for high-throughput prediction and sophisticated quality control applications.
Optimization Algorithms	Bayesian Optimization (BO), ACO	Reaction Conditions, Dye Recipes, Catalyst Use	Used for finding global optima in complex manufacturing or synthesis steps, crucial for enhancing efficiency and quality control.
Inverse Design Models	PolyBART, PolyID	Thermal Stability, Fire Resistance, Specific Molecular Structure	Generative design based on desired output properties, fundamentally reversing the traditional synthesis-first R&D workflow.

## 9 Mechanism-Aware and Physics-Informed Learning for Stimuli-Responsive Polymers: Governing Equations, Implementation, and Validation

Stimuli-responsive polymer behavior is essentially characterized by coupled transport, thermodynamics, and mechanics, hence “physics-informed” modeling should be based on the equations that describe such systems. In the context of scientific machine learning, physics-informed neural networks (PINNs) provide one concrete route: they train neural surrogates while penalizing violations of the underlying PDE/ODE constraints via residual terms in the loss function. This allows for data-efficient learning and parameter discovery under physically consistent dynamics.^118^ In release and delivery, stimuli responsiveness is commonly manifested by diffusion-reaction coupling (e.g., diffusion with degradation, binding/unbinding, or stimulus-triggered conversion), in which concentration fields evolve under conservation laws and boundary-driven mass transfer. Classical diffusion frameworks (and their drug-release adaptations) provide mechanistic baselines that can be combined with machine learning to learn unknown constitutive closures such as concentration-dependent diffusivity or stimulus-dependent source terms while enforcing conservation and boundary/initial conditions.^119^ The equilibrium behavior of swelling-driven actuation and shape change is determined by polymer network thermodynamics (Flory–Rehner-type treatments), whereas transients are controlled by poroelastic/chemo-mechanical swelling kinetics, where solvent transport links substantial deformation.^120^ Mechanism-aware learning can thus be implemented as (i) a hybrid model that preserves the free-energy/constitutive structure and uses ML only to infer uncertain parameters (e.g., interaction parameter, permeability, effective modulus), or (ii) a PINN that enforces the coupled field equations while fitting swelling ratio, curvature, or strain-time data. Thermodynamically consistent continuous frameworks for gels (coupled diffusion + large deformation; permeation + large deformation) provide a solid physical basis for such applications.^121^ Responsiveness in LCST/UCST-type transitions is obtained from polymer–solvent (or polymer–polymer) phase behavior controlled by Flory–Huggins thermodynamics and composition/temperature-dependent interaction parameters, with PNIPAM acting as a typical LCST example.^122^ Physics-aware machine learning can constrain predictions to be consistent with phase-diagram structure (for example, coexistence bounds), as well as learn system-specific mappings across composition, additives, and temperature. Finally, because “physics-informed” can otherwise read as aspirational, explicit validation against data-driven baselines is essential: models should be compared on identical splits using both accuracy metrics and physics-consistency measures (e.g., PDE residual norms, conservation error), and they should be stress-tested under extrapolation (new thickness/geometry, temperature range, or boundary conditions), where physics constraints are expected to yield the most significant advantage.^118^

### 9.1 Bridge between AI/ML and Stimuli-Responsive Polymeric Materials

To connect the review’s scope with the breadth of stimuli-responsive materials and device demonstrations, it is critical to balance polymerization/automation workflows with the key responsive classes that dominate real-world actuation, sensing, and adaptive-device research. Multistimuli and logic-type materials are prime instances of breadth that goes beyond synthetic control. Multiresponsive particles, films, and hydrogels have been extensively studied, including structures that incorporate orthogonal cues (pH, temperature, light, and redox) and allow for programmable behaviors like gating or multistage release.^123^ ML is commonly utilized in these systems to learn multidimensional stimulus-response surfaces and establish the best operating regimes for selectivity and cross-talk minimization. Electroactive polymers (EAPs) are a popular device category whose performance is influenced by electromechanics and manufacturing constraints. Dielectric elastomer actuators (DEAs) have become indispensable in soft robotics due to their high strain and rapid response, whereas device-level issues include electrode patterning, breakdown, and cyclic reliability.^124^ According to recent research, learning-based pipelines may autonomously generate complicated electrode patterns using neural-network surrogates, as well as optimize and experimentally verify them, resulting in a concrete, device-facing AI method. An Automated Design Framework for Dielectric Elastomer Actuators: Neural Network-Based Real-Time Simulation, Genetic Algorithm-Based Electrode Optimization, and Experimental Validation. Magneto-responsive composites and magnetoactive metastructures provide untethered actuation and shape morphing, but their design space (geometry, magnetization, and field) is multidimensional. A excellent example is topology-optimization-based inverse design of magneto-active metasurfaces/robots to obtain desired 3D morphologies and motion modes—an method that is closely related to “automation” but focuses on device functionality rather than polymerization.^125^ Mechanophore-based systems are another form of responsive system that uses mechanical force as a “stimulus” and outputs optical/chemical reporting or activation. Spiropyran mechanophores, for example, have been used to quantitatively map stresses in elastomers, and larger studies emphasize their potential for damage detection and mechanochemical sensing.^126^ These technologies allow ML to connect molecular structure/network architecture to force-response signatures and comprehend large imaging/spectroscopy data sets during deformation. In Table 3 we have summarized recently published articles focusing on AI and ML integrated Stimuli-Responsive polymers. Finally, to address long-term cycling/aging, we explicitly mention durability as a first-class topic: DEAs and similar EAP devices exhibit fatigue and breakdown phenomena under cyclic electromechanical loading, underscoring the necessity for lifetime-aware design and data-driven dependability prediction.^127^ This section rebalances the review by combining improvements in synthesis/automation with equally extensive treatment of device-relevant stimuli-responsive material classes and operational restrictions.

**Table 3:** Summary of Recently Published Articles Focusing on AI and ML Integrated Stimuli-Responsive Polymers

Year	Polymer system/material class	Stimulus	AI/ML objective	ML method
2018 ^128^	poly(*N*-isopropylacrylamide) (PNIPAM)	Temperature change drives the coil-to-globule transition of thermosensitive PNIPAM in aqueous solution.	Learn low-dimensional embeddings of polymer conformations to identify states and pathways in the coil-to-globule transition.	Nonmetric Multidimensional Scaling (NMDS) applied to coarse-grained MD trajectory data (distance matrices between conformations).
2019 ^24^	poly(2-oxazoline)	Polymer composition and molecular weight determining cloud point of poly(2-oxazoline)s in water.	Forward: predict cloud point from polymer structure (monomer counts, type, molecular mass). Inverse: design polymers with target cloud point.	Gradient Boosting Regression with decision trees (forward model), plus particle swarm optimization (inverse design) and an ensemble of neural networks for candidate filtering.
2020 ^129^	poly(NIPAAm-*co*-AAc)-PEG interpenetrating polymer network (IPN) hydrogel	pH and temperature changes controlling DOX release from poly(NIPAAm-*co*-AAc)/PEG IPN hydrogel.	Predict cumulative doxorubicin release (%) as a function of time, pH, and temperature.	Artificial Neural Networks (ANN), Least-Squares Support Vector Machine (LS-SVM), and Support Vector Regression (SVR); ANN performed best.
2020 ^26^	Elastin-like polypeptides (ELP) and ELP–PEI copolymers	Solution conditions: salt concentration, pH, polymer concentration, and polymer type affecting LCST transition and aggregation of ELP and ELP-PEI copolymers.	Predict transition temperature T_T_ and hydrodynamic radius *R*_h_ of ELP and ELP-PEI copolymers from solution parameters.	Five supervised regressors: Multiple Linear Regression, Elastic-Net Regression, Support Vector Regression (SVR), Multi-Layer Perceptron (MLP), and Random Forest; MLP gave the best accuracy.
2021 ^130^	Poly(*N*-isopropylacrylamide-*co*-methoxy triethylene glycol acrylate) (NIPA-*co*-MTGA)	Salt type and concentration, and NIPA/MTGA composition controlling LCST of NIPA-*co*-MTGA in aqueous salt solutions.	Predict LCST of NIPA-*co*-MTGA and related polymers from salt properties and polymer composition	Support Vector Regression (SVR) with kernel functions, the first application of SVR to LCST prediction in salt solutions.
2021 ^131^	Thermoset shape-memory polymers (TSMPs) (epoxy networks)	None in the physical sense; “stimulus” is the target design space of UV-curable TSMPs	Learn a latent representation of TSMP-like chemistries and generate new TSMP formulations with high recovery stress and moderate *T*_g_.	Transfer-learning Variational Autoencoder (VAE) pretrained on drug molecules, combined with a weighted-vector composition scheme; outperforming SVM baselines.
2022 ^132^	Hydrophobic comonomer content and chemistry (DMA-*co*-RA composition) controlling cloud point *TCP* in water.	Thermoresponsive Behavior	Predict cloud point T_CP_ of P(DMA-*co*-RA) copolymers from computed hydrophobicity descriptor Log Poct/SA.	Simple linear regression (no machine learning model beyond linear fit between Log Poct/SA and T_CP_).
2023 ^133^	*poly-*N*-isopropylacrylamide (PNIPAM)*	Solution conditions (polymer structure via PFP, concentration, salt, pH, etc.) governing LCST/cloud-point behavior of thermoresponsive polymers.	Predict cloud-point/LCST temperature of a wide variety of LCST polymers from the polymer fingerprint plus solution descriptors.	Deep feed-forward Artificial Neural Network in PyTorch, taking the polymer fingerprint (PFP) as input and trained with Adam on MSE; FP size and hyperparameters tuned by random search.
2024 ^28^	*PNIPAAm*,	Temperature change (swelling vs nonswelling) of PNIPAAm-based hydrogels, encoded via synthesis and test parameters (monomer/cross-linker content, solvent, pH, etc.).	Predict discrete swelling states (swelling degree at several temperatures) of temperature-responsive hydrogels directly from synthesis and swelling-test setup parameters.	Feed-forward Artificial Neural Networks (multiple variants; final model: 4 hidden layers with 128–64–32–16 nodes, tanh activation, Adam optimizer, L2 regularization).
2025 ^30^	pH-responsive hydrogels (continuum swelling dynamics)	pH change driving transient swelling and stress evolution in pH-responsive hydrogels.	Predict spatiotemporal hydration (swelling) and stress distributions from time and material/geometry parameters.	Two-step augmented neural network: first ANN predicts hydration, second ANN takes predicted hydration to output radial and hoop stresses.

## 10 Predictive Models for Performance Assessment

Physics- and knowledge-informed machine learning is quickly becoming a strong way to test the performance of polymers and biomaterials. This is because it solves long-standing problems with the “black box” nature of traditional neural networks in safety-critical areas like predicting toxicity and biocompatibility. Physics-informed neural networks (PINNs) provide better interpretability, extrapolation, and data efficiency by incorporating domain equations and physical constraints into data-driven models. This helps close the gap between conventional polymer science and contemporary AI-based predictive modeling.^79^

### 10.1 Predictive AI for Polymer Performance and Safety

Applied machine learning has already demonstrated substantial value in predicting polymer properties such as mechanical strength, glass transition temperature, rheology, and degradation behavior from high-dimensional structural and processing descriptors. These models enable rapid screening of candidate formulations and process conditions, reducing experimental workload and accelerating iterative design cycles, particularly in areas like structural composites, hydrogels and scaffold materials for biomedical applications. In parallel, toxicology and safety assessment have seen a shift from purely empirical quantitative structure–activity relationship (QSAR) models to deep learning architectures that can capture complex nonlinear relationships in chemical space, offering improved balanced accuracy for end points such as drug-induced liver injury (DILI), carcinogenicity, and mutagenicity.^80,81^

Nevertheless, many polymer scientists and toxicologists remain reluctant to fully embrace deep neural networks because their internal representations are difficult to interpret in terms of established physical or chemical principles. For polymer systems, where properties depend on a hierarchy of features from monomer chemistry to chain architecture, morphology and processing history, such opacity raises legitimate questions about generalizability, failure modes, and the extent to which predictions respect known conservation laws or constitutive behaviors. This trust deficit is especially acute for safety-critical decisions in implantable biomaterials or regulatory toxicology, where interpretability and mechanistic plausibility are valued as highly as raw predictive performance.^82^

### 10.2 Physics-Informed Neural Networks for Polymers

Recent reviews on “Physics-Informed Neural Networks in Polymers” describe how PINNs explicitly encode governing equations—such as balance laws, constitutive models, diffusion–reaction kinetics, or phase-field formulations—directly into the loss function of neural networks. Instead of learning unconstrained mappings from inputs to outputs, the network is penalized whenever its predictions violate the residuals of partial differential equations (PDEs) or other physical relationships, which enforces physically consistent behavior even in regions of sparse or noisy data. Consequently, PINNs can interpolate between limited experimental measurements and extrapolate into new regimes (e.g., loading rates, temperatures, molecular weights) while still respecting thermodynamics and mechanics, something that purely data-driven models often fail to achieve.^83^

Concrete polymer applications illustrate this advantage. A physics-enforced neural network has been proposed to predict polymer melt viscosity across broad ranges of shear rate, temperature, and molecular characteristics by embedding rheological scaling and flow equations into the learning objective, achieving better generalization than classical ANNs and Gaussian processes when training data are limited. Other studies have coupled PINNs with phase-field models to design high-energy-storage polymer nanocomposites, where the network learns microstructure–property relationships under the constraint of phase-field evolution equations, thereby enabling more reliable predictions of dielectric response and breakdown strength. Similar strategies have been applied to thermo-mechanical loading of fiber-reinforced polymers under laser irradiation, where PINNs capture surface temperature evolution and damage initiation by satisfying heat conduction and boundary-condition constraints, which is difficult to accomplish with black-box networks alone.^84^

## 11 AI for Toxicity, Biocompatibility, and Regulatory Initiatives

In toxicology, Li et al.^134^ have developed a family of deep learning models—DeepDILI, DeepCarc and DeepAmes—that use model-level ensemble representations to predict DILI, carcinogenicity and Ames mutagenicity, consistently outperforming conventional QSAR and ensemble baselines. These models exploit complementary strengths of multiple base learners and deep architectures to capture subtle structure–toxicity patterns across diverse chemical spaces, thereby supporting earlier and more reliable identification of hazardous compounds in drug discovery pipelines. Building on this work, the US Food and Drug Administration’s National Centre for Toxicological Research has launched the SafetAI Initiative, which aims to integrate such deep learning frameworks into regulatory workflows for IND review, enabling the prediction of key toxicological end points (e.g., hepatotoxicity, carcinogenicity, mutagenicity, nephrotoxicity, cardiotoxicity) directly from molecular structure and related descriptors before human exposure.

Beyond small-molecule drugs, applied machine learning is increasingly recognized as a driver for polymeric biomaterials design, particularly for predicting cytotoxicity, immunogenicity and biocompatibility of novel polymer scaffolds and hydrogels. However, existing data sets for biomedical properties are often small, noisy and heterogeneous due to nonstandardized testing protocols, complicating the application of purely data-hungry deep networks. This context makes PINNs and related “scientific machine learning” particularly attractive, as they can incorporate degradation kinetics, diffusion-controlled release, and mechanics of tissue–material interactions as physics-based priors, thereby extracting more value from limited experimental data while enhancing mechanistic interpretability.^85^

## 12 Integrated Perspective and Outlook

Taken together, these developments suggest that physics-informed and hybrid neural network architectures can substantially alleviate the “black-box” criticism that has limited the adoption of AI in polymer science and biomaterials safety assessment. By unifying data-driven pattern recognition with explicit physical and chemical knowledge, PINNs provide a route to predictive models that are not only accurate and data-efficient, but also aligned with the conceptual frameworks familiar to polymer chemists, materials engineers and toxicologists. When combined with regulatory-oriented initiatives such as the FDA’s SafetAI program and deep toxicity frameworks like DeepDILI and DeepCarc, these approaches point toward an ecosystem in which AI can reliably guide the design of safer, more effective polymers and drugs, while maintaining the transparency and scientific rigor demanded in high-stakes biomedical applications.^85^

## 13 Biomedical Applications of Stimuli-Responsive Polymer Based on AI and ML

Artificial intelligence and machine learning (AI/ML) are changing biomedical polymer and biomaterial research by allowing for quantifiable correlations between structure, interactions, and biological function has been shown in Figure 8. Chemical composition, topology, architecture, chirality, and sequencing all contribute to physicochemical behavior at the molecular and material levels, which determines biological responses such as bioavailability, toxicity, and therapeutic efficacy. AI/ML offers a cohesive framework for learning these highly nonlinear structure–property-performance correlations and guiding rational and inverse design. In drug distribution, machine learning (ML) has emerged as a potent technique for predicting formulation performance, release kinetics, and in vivo relevance. Supervised learning methods trained on formulation descriptors have been demonstrated to reliably predict long-acting drug release profiles, allowing inverse design of polymer-drug systems with targeted pharmacokinetics, greatly decreasing experimental iterations.^135^ Recent studies further highlight how ML enhances nanoparticle and polymeric administration through formulation optimization, stability prediction, and ADMET-relevant end points, taking the field beyond empirical screening.^136^ The nanobio interface and interactions panel in your figure is extremely relevant to current AI-driven research. Protein adsorption and corona formation determine nanoparticles’ biological identity, which influences cellular uptake, biodistribution, and immune response. Machine learning algorithms accurately predicted the protein corona composition from nanoparticle physicochemical characteristics, suggesting that complicated biological interactions may be predicted computationally.^137^ More recently, curated community data sets and databases have allowed large-scale ML meta-analysis of nanobio interactions, boosting model robustness and interpretability.^138^ For 3D printing and bioprinting, AI/ML solves one of the primary bottlenecks: the multidimensional parameter space that connects ink composition, rheology, cross-linking chemistry, and printing circumstances to print quality and biological consequences. Data-driven models have been used to forecast printability, strand stability, and shape accuracy, as well as to optimize printing parameters based on biological restrictions such as cell viability. Recent reviews highlight how ML is currently integrated into biofabrication workflows for parameter optimization, quality prediction, and adaptive control.^139^ The biomarker and image analysis component reflects one of the most mature biomedical ML domains. Deep learning architectures are currently the industry standard for medical image reconstruction, segmentation, object detection, and enhancement, allowing for the quantitative extraction of imaging biomarkers to guide diagnosis and therapy. Large-scale surveys demonstrate that convolutional and transformer-based models outperform classical methods in terms of robustness and accuracy for biological picture segmentation and analysis.^140^ Complementary studies focus on data-centric AI methods, such as augmentation and uncertainty modeling, to increase generalization in clinical imaging workflows. Overall, it accurately describes the current paradigm: AI/ML serves as a translational engine that links material structure to biological interaction, treatment performance, and clinical outcomes. AI/ML accelerates the creation of next-generation biomedical polymers and biomaterials for drug delivery, biofabrication, biomarker discovery, and image-guided therapy by combining property prediction, optimization, high-throughput screening, and inverse design.

**Figure 8:**
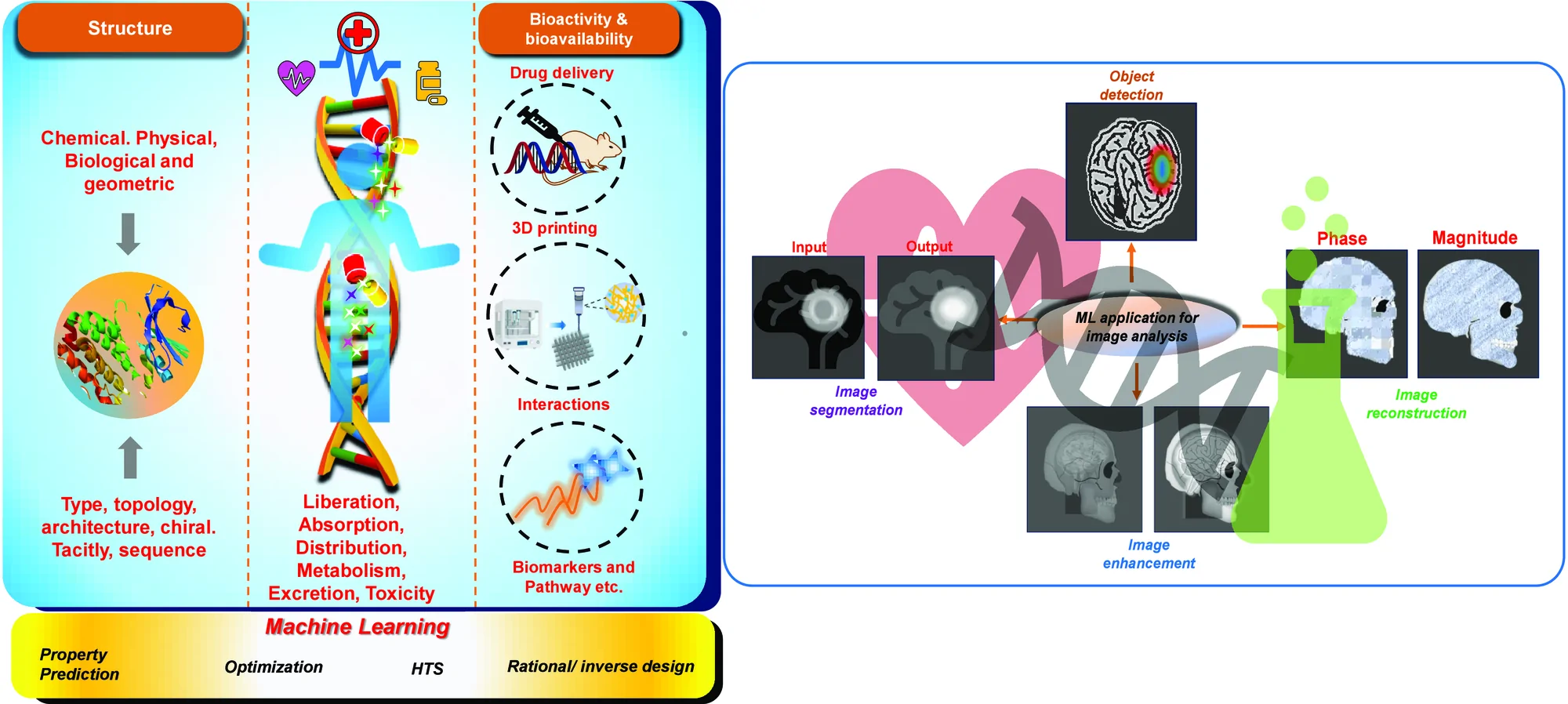
Machine-learning–enabled structure–bioactivity–function relationships and image-based analysis in biomedical materials design. Reproduced with permission.^99^ Xie et al. Copyright 2025, John Wiley and Sons, Creative Commons CC BY license, open access.

## 14 Recent Progress of Stimuli-Responsive Polymeric Materials with AI ML Formulation

Polymer materials often have a wide range of molecular weights, complicated three-dimensional conformations, branching and cross-linking structures, and interchain entanglements. Because of their intrinsic structural intricacies, it is extremely difficult to precisely capture microscopic information and translate them to macroscopic attributes.

The issues with descriptors primarily center on the following three aspects: (1) Given the diversity of polymer structures, it is difficult to generate effective descriptors; (2) high-dimensional descriptors are prone to the “curse of dimensionality”; and (3) descriptor sources and scales differ significantly, making integration challenging. In less than ten years, the paradigm of machine learning approaches gained widespread use in numerous polymer systems and amassed over 4000 scholarly publications. These could include the development of long-lasting polymers for engine combustion, Energy^141^ long-lasting coatings to withstand various stresses. These have also extended to the design and optimization of cutting-edge materials; two common examples are the creation of superhydrophobic polymer surface topographies with maximum water contact angle and Laplace pressure^142^ and the screening of unique microstructures to convert brittle polymers into supercompressible meta-materials.^143^ In addition to the above-mentioned areas, the advancement of machine learning techniques used in polymer science is still updated on a regular basis. For a brief overview, they tabulated some recent representative results, taking into account the polymers, functionalities, key experimental parameters related to targets for prediction and interpretation, and the machine learning algorithms used in the modeling. Most of them fit into one or more of these categories. Regression (Reg) and classification (Cla) account for the majority of machine learning modeling, while a small number of studies focus on clustering, dimension reduction, and interacting with experiments for iterative optimization. To improve the expressiveness and adaptability of material descriptors, two complementary approaches have been advanced: multiscale modeling and time-series analysis. Materials undergo dynamic changes throughout service, such as thermal aging, mechanical fatigue, and environmental exposure. Therefore, developing time-dependent descriptors is critical. Sequence-based deep learning architectures, including long short-term memory (LSTM) networks and Transformers, have been used to track material property evolution over time. These strategies improve the time-resolution of descriptors and create new paradigms for predicting nonequilibrium behavior, service life, and durability. Lee et al. used feed-forward neural network (FNN) and LSTM models to predict the tensile behavior of polymer matrix composites (PMCs), demonstrating high accuracy in forecasting tensile failure spots.^144^ Despite these significant developments in the field (Figure 9), substantial problems remain, including data uncertainty, handling high-dimensional data, and removing redundant characteristics.

**Figure 9:**
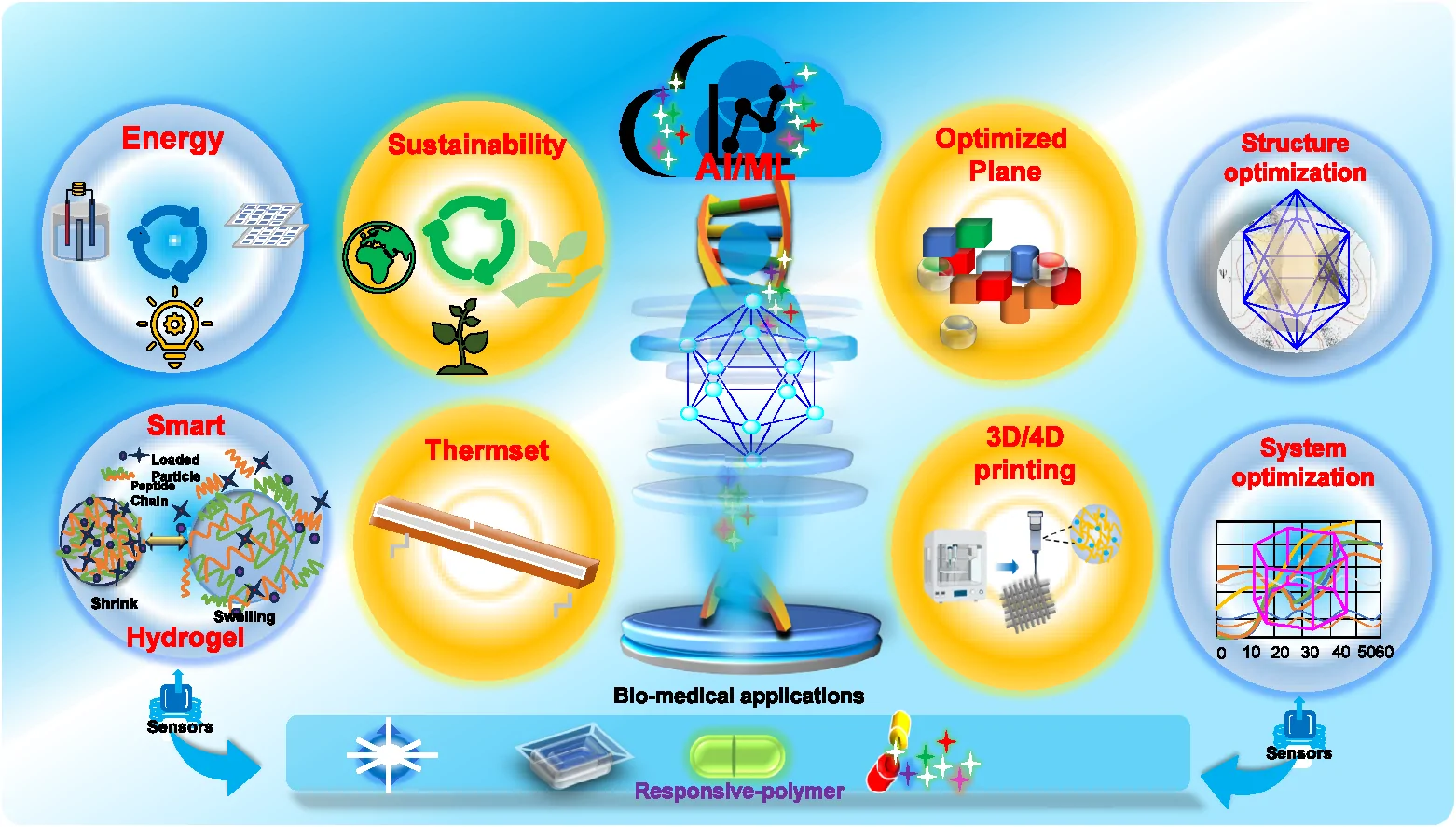
AI/ML-driven design ecosystem for smart, sustainable, and application-ready responsive polymer systems.

### 14.1 Critical Challenges and Best Practices

AI-based stimuli-responsive polymer models have shown promise, but changing them from academic curiosity to reliable engineering tools requires addressing representation, data quality, and validation issues. We answer practitioner issues by synthesizing recent literature insights. By substituting predictive Quantitative Structure–Property Relationship (QSPR) models that link chemical designs to particular environmental triggers for conventional experimental iterations, artificial intelligence (AI) and machine learning (ML) are transforming the design of stimuli-responsive polymers (SRPs). By determining the quantitative connections between dynamic response behavior, environmental factors, and polymer structure. The stimulus threshold, reversibility, and response time constants related to stimuli-induced transitions can be predicted by training machine learning models on data sets that include molecular descriptors like monomer composition, functional groups, hydrophilicity, molecular weight, and cross-link density in addition to experimental response data. Researchers can predict p*K*_a_-driven phase transitions in pH-sensitive hydrogels for targeted drug delivery^145^), optimize chromophore isomerization for light-activated actuators^146^ and precisely tune the Lower Critical Solution Temperature (LCST) for thermoresponsive systems like Graph Neural Networks and Bayesian optimization. By handling high-dimensional data, such as cross-linking densities and monomer sequences represented by SMILES strings, these computational tools make it possible to find multiresponsive materials that respond to intricate biological or environmental cues with previously unheard-of speed and precision^147^ Beyond threshold prediction, machine learning frameworks are being investigated more and more to evaluate stimulus-response data sets and correlate polymer architecture with fatigue resistance, cycling stability, and hysteresis behavior. This allows for the logical design of long-lasting stimuli-responsive materials for use in drug delivery, sensing, and smart systems^148,149^

#### 14.1.1 Limitations

A brief introduction has been given to published research and the development trend pertaining to applications of ML in polymer materials in recent years.

Additionally, case examples were utilized to summarize the ML workflow and frequently used algorithms and descriptors in the design and discovery of polymer materials. However, ML applications in the polymer area are still in their early stages and have a lot of restrictions. The qualities of materials that fall outside of the recognized range of their values are the focus of many crucial design issues. Because machine learning models are by nature interpolative, they are often less trustworthy when looking for extreme possibilities^150^ It is necessary to overcome a number of practical obstacles pertaining to molecular representation, data quality, and rigorous model validation in order to translate the methods from academic investigations into trustworthy engineering tools. While the availability of high-quality, standardized experimental data sets is crucial for capturing complex structure–property relationships and dynamic response behaviors, accurate representation of polymer structures and stimuli-dependent features is still necessary for developing predictive machine learning models. In order to facilitate the development of more dependable AI-driven frameworks for the design of stimuli-responsive polymer systems, this work synthesizes insights from recent literature to highlight important practitioner-oriented considerations and outlines strategies to improve data curation, descriptor selection, and validation practices.

### 14.2 Which Responsive System Representations Work Best?

Complex macromolecular structures are difficult to translate into machine-readable inputs in polymer informatics. Differences between static and learnt descriptors Traditional molecular fingerprints (Morgan, MACCS) typically miss long-range order and topology needed for stimuli-responsiveness. Sequence-based representations (SMILES/SELFIES) linked with Recurrent Neural Networks (RNNs) or Transformers capture sequence-property relationships better for linear copolymers (e.g., sequence-controlled PNIPAM), for 3D cross-linked networks like hydrogels. Graph Neural Networks (GNNs) are better because they model the polymer network’s architecture and connections rather than considering it as a linear string.

### 14.3 Solving “Small Data”

Unlike image recognition, polymer science rarely provides “Big Data.″ Data sets are typically noisy, sparse, and tiny (*N* < 1000). Instead of collecting enormous random data sets, practitioners should use Active Learning loops. An acquisition function can select the subsequent trial that maximizes information gain, allowing researchers to optimize responsive qualities like swelling ratio with 80–90% fewer experiments than random screening. Transfer Learning: Pretraining models on large generic polymer databases and fine-tuning on the experimental data set have worked well for specialized responsive polymers with limited data.

### 14.4 Robust Validation

Beyond Random Splits, excellent R2 scores on random test splits can be deceiving due to polymer families’ remarkable structural similarity. Random splitting typically leads to “memorization” rather than learning since train and test sets contain chemically identical analogues. (Use Scaffold or Time-Based Splitting. Models should be evaluated on different chemical scaffolds or processing settings than during training to assess generalizability and transferability across polymer chemistries.)

### 14.5 Metrics for “Responsive” Function

Mean absolute error (MAE) and root mean squared error (RMSE) measure static accuracy but not dynamic performance. We recommend device-relevant metrics for stimuli-responsive systems: Switching Prediction accuracy: Error margin in crucial transition point prediction.

## 15 Conclusions and Future Objectives

AI has shown promising results in polymer research, such as molecular structure design, property prediction, process parameter optimization, and the creation of intelligent closed-loop laboratories. These advancements are gradually shifting the focus of materials research from experience to data. However, compared to other material systems, the application of AI in polymers is still in the early experimental stages. The present issues include (1) the lack of large-scale, representative, high-quality, structured data sets, (2) the absence of universal descriptor systems that can accurately depict the multiscale structures of polymers, and (3) the requirement for model architecture and training mechanism optimization. Smart stimuli-responsive AI and ML techniques show promise as a flexible and effective means of comprehending, identifying, and improving scientific and technological issues in polymer research. They have amassed a wealth of information, tactics, and algorithms that can help establish a new paradigm. When machine learning techniques are applied to well-defined targets, their numerical values or enumerable classes are spread in a finite range within the data set’s boundaries, and the targets have explicit or implicit correlations with the features or attributes. Depending on the techniques and algorithms used in data and feature engineering, an ML-based model is considered credible when the correlations demonstrate adequate confidence, typically in regression, classification, or clustering. The maturity of this new paradigm is demonstrated throughout the essay, emphasizing its wide adaptability to address various issues and challenges in polymer research. In terms of their future development, the following paths show great promise for elevating this new paradigm to a new level in the advancement of polymer research.
